# Supplementary material for: Enteric virome negatively affects seroconversion following oral rotavirus vaccination in a longitudinally sampled cohort of Ghanaian infants
Source: Cell Host Microbe. 2022 Jan 12;30(1):110–123.e5. doi: 10.1016/j.chom.2021.12.002 (PMC8763403; doi:10.1016/j.chom.2021.12.002)
Supplement: Document S1. Figures S1–S6 and Table S1 [file mmc1.pdf]

Cell Host & Microbe, Volume *30*

Supplemental information

Enteric virome negatively affects seroconversion following oral rotavirus vaccination in a longitudinally sampled cohort of Ghanaian infants

Andrew HyoungJin Kim, George Armah, Francis Dennis, Leran Wang, Rachel Rodgers, Lindsay Droit, Megan T. Baldridge, Scott A. Handley, and Vanessa C. Harris

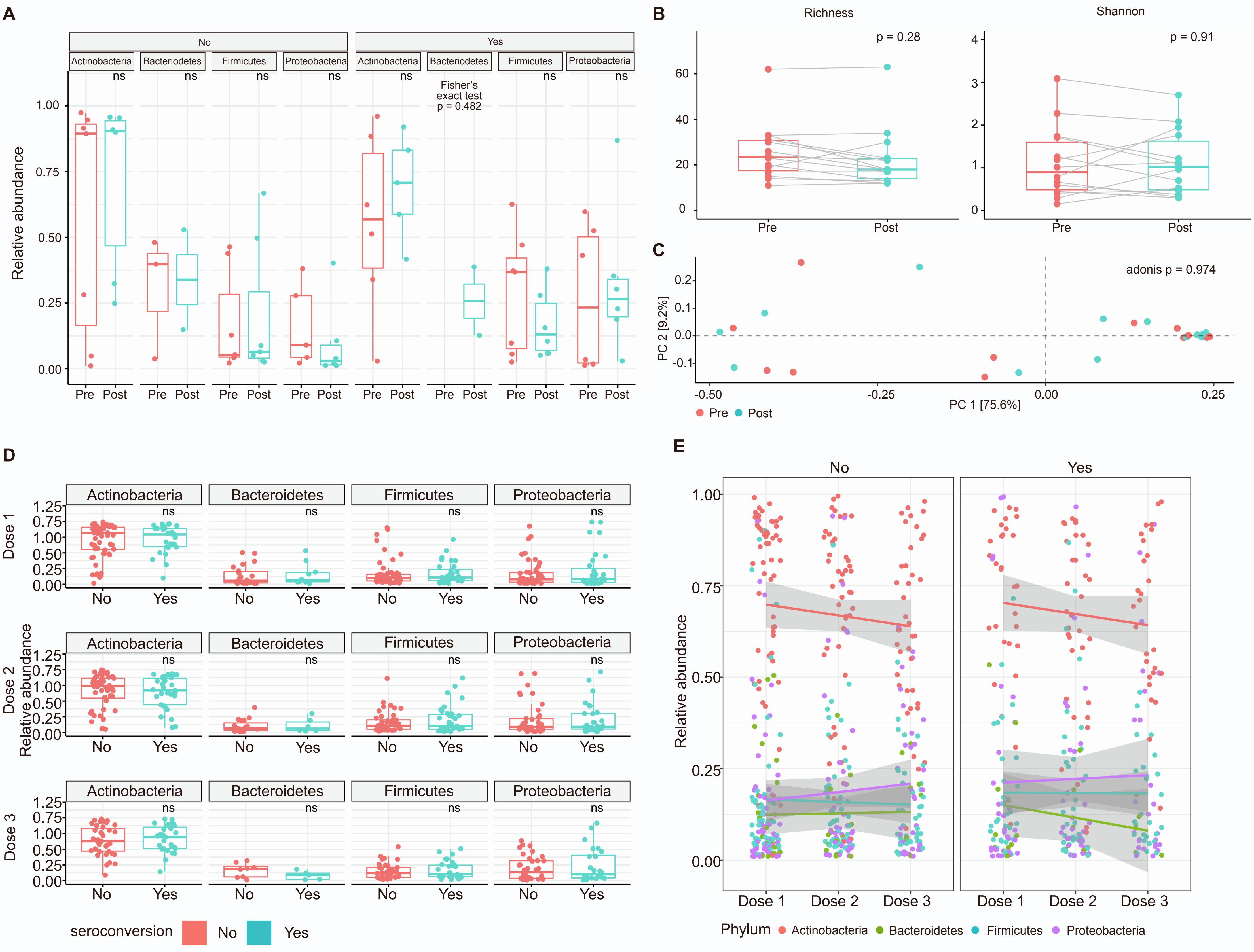

Figure S1. Phylum-level bacteriome composition analysis shows no differences between pre- and post- vaccination samples and specific to seroconversion, related to Figure 2. A) The relative abundance of bacterial phyla in 14 matched pre- and post-vaccination samples at dose 1, **B)** Bacterial richness and diversity between pre- and post-vaccination samples at dose 1, **C)** Bacterial beta diversity (weighted UniFrac distance) of pre- and post-vaccination samples at dose 1. Wilcoxon test and Permutational Multivariate Analysis of Variance (ADONIS) were used to compare between serostatus groups in alpha- and beta-diversity analyses, respectively. **D)** The relative abundance of bacterial phyla in Ghanaian infants identified as non-seroconverters ("No") and seroconverters ("Yes") at three doses (Dose 1, 2, 3) and **E)** change over time. Lines depict the linear model while greyed areas indicate the 95% confidence level interval of the model. Statistical comparison between two serostatus groups was performed using Wilcoxon test (ns = not significant). n = 148 averaged non-seroconverter samples (Dose 1: 60, Dose 2: 49, Dose 3: 39) and 99 averaged seroconverter samples (Dose 1: 35, Dose 2: 35, Dose 3: 29).

Dose	ASV	lfc	lfcse	adj p-value	Sequence	Kingdom	Phylum	Class	Order	Family	Genus	Species	Pearson's rho
Dose 1	3	2.845	0.736	3.63E-03	GCAAGCGTTAATCGGAATT	Bacteria	Proteobacteria	Gammaproteobacteria	Enterobacteriales	*Enterobacteriaceae*	NA	NA	DA
Dose 2	4	2.640	0.632	5.99E-04	CCGAGCGTTGTCCGGATTT	Bacteria	Firmicutes	Bacilli	Lactobacillales	*Streptococcaceae*	*Streptococcus*	NA	DA
Dose 2	5	9.194	2.326	1.18E-03	CCGAGCGTTGTCCGGATTT	Bacteria	Firmicutes	Bacilli	Lactobacillales	*Streptococcaceae*	*Streptococcus*	NA	DA
Dose 2	6	-9.932	2.118	8.33E-05	CCGAGCGTTGTCCGGATTT	Bacteria	Firmicutes	Bacilli	Lactobacillales	*Streptococcaceae*	*Streptococcus*	NA	DA
Dose 2	45	6.292	1.234	2.08E-05	GCAAGCGTTATCCGGATTT	Bacteria	Firmicutes	Bacilli	Lactobacillales	*Lactobacillaceae*	*Lactobacillus*	*fermentum*	DA
Dose 3	3	3.047	0.865	1.39E-02	GCAAGCGTTAATCGGAATT	Bacteria	Proteobacteria	Gammaproteobacteria	Enterobacteriales	*Enterobacteriaceae*	NA	NA	DA
Dose 3	32	2.235	0.716	3.91E-02	GCGAGCGTTGTCCGGAATT	Bacteria	Actinobacteria	Actinobacteria	Actinomycetales	*Micrococcaceae*	*Rothia*	*mucilaginosa*	DA
Dose 3	92	0.995	0.512	4.84E-03	GCGAGCGTTATCCGGATTC	Bacteria	Actinobacteria	Actinobacteria	Coriobacteriales	*Coriobacteriaceae*	*Eggerthella*	*lenta*	0.453

lfc	log2(Fold change)
lfcse	log2(Fold change Stardard Error)
NA	Taxonomy not assigned
DA	Discrete analysis

Table S1. Summary table of bacterial markers identified by DESeq2 analysis at each dosing period, related to Figure 2. Identification of bacterial markers was performed using DESeq2 and multiple Pearson’s correlation analyses with cut-off threshold of adjusted p value < 0.05. Adjusted p values were attained for each bacterial marker by performing Wald test followed by correction using Benjamini and Hochberg method for DESeq2 and Pearson’s correlation analysis followed by correction using Bonferroni method. Log2(Fold change) and log2(Fold change Standard Error) show the seroconverter (“Yes”) over non-seroconverter (“No”) fold change and its standard error for the abundance of each bacterial marker. Black texts indicate markers selected from DESeq2 analyses and gray texts indicates a marker selected from multiple Pearson’s correlation analysis.

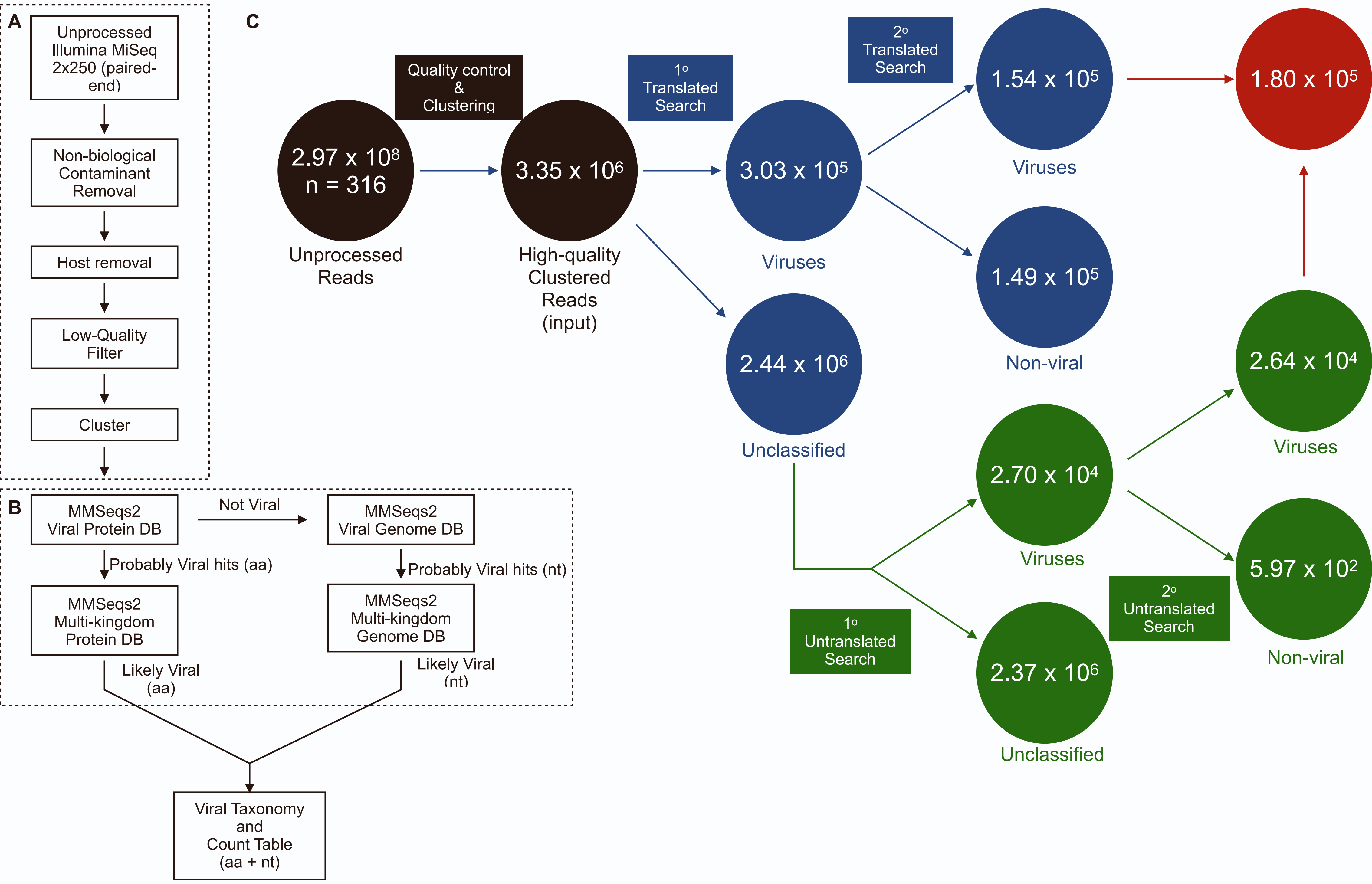

Figure S2. Viral taxonomic analysis of Illumina reads, related to STAR Methods. High-quality reads from 316 samples (black circle) were quality controlled to remove non-biological sequence (primer, adapter), host and low-quality sequence (Figure S2). These high-quality sequences were 6-frame translated and queried (blue path) against a viral protein sequence database (UniProt viral proteins clustered at 99% identity) followed by a secondary translated query of all potential viral sequences against a universal protein sequence database (UniClust30) to remove mis-classified viral sequences (Mirdita et al., 2017). Sequences that were not classified in the translated search were queried (green path) against a viral reference genome database (all RefSeq viral genomes plus their nearest neighbors). Potential viral sequences were subsequently queried against a bacterial plus viral genome database to remove sequences ambiguous to bacteria and virus. The translated and untranslated query sequences assigned to a viral taxonomic lineage were combined into a final (red circle) sequence table.

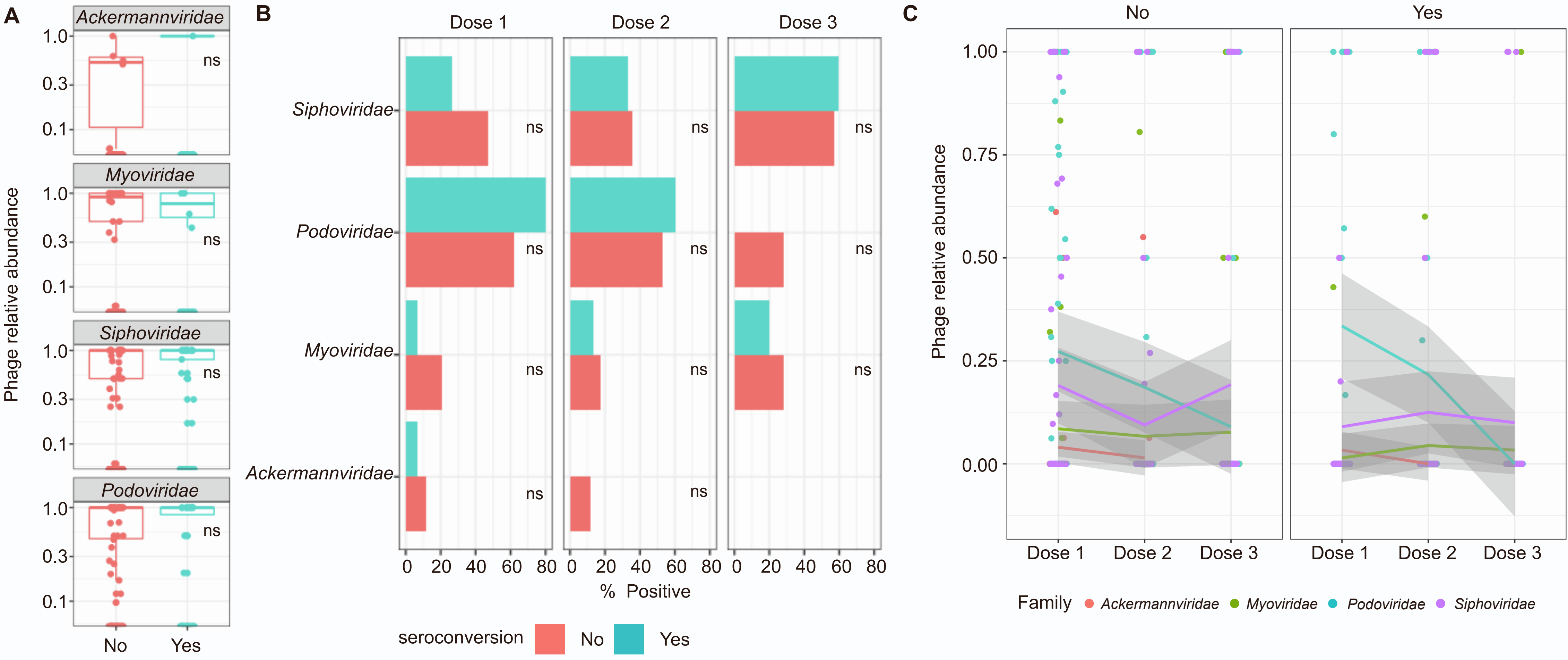

Figure S3. Phage composition does not associate with seroconversion when analysed at the family-level, related to Figure 3. A) phage relative abundance in Ghanaian infants identified as non-seroconverters ("No") and seroconverters ("Yes") at three doses (Dose 1, 2, 3). **B)** The percent positive of phage families at three doses (Dose 1, 2, 3). **C)** phage relative abundance change over time. Statistical comparison between two serostatus groups was performed using Wilcoxon test (ns = not significant). Lines depict the linear model while greyed areas indicate the 95% confidence level interval of the model. n = 216 non-seroconverter samples (Dose 1: 54, Dose 2: 84, Dose 3: 78) and 162 seroconverter samples (Dose 1: 30, Dose 2: 72, Dose 3: 60).

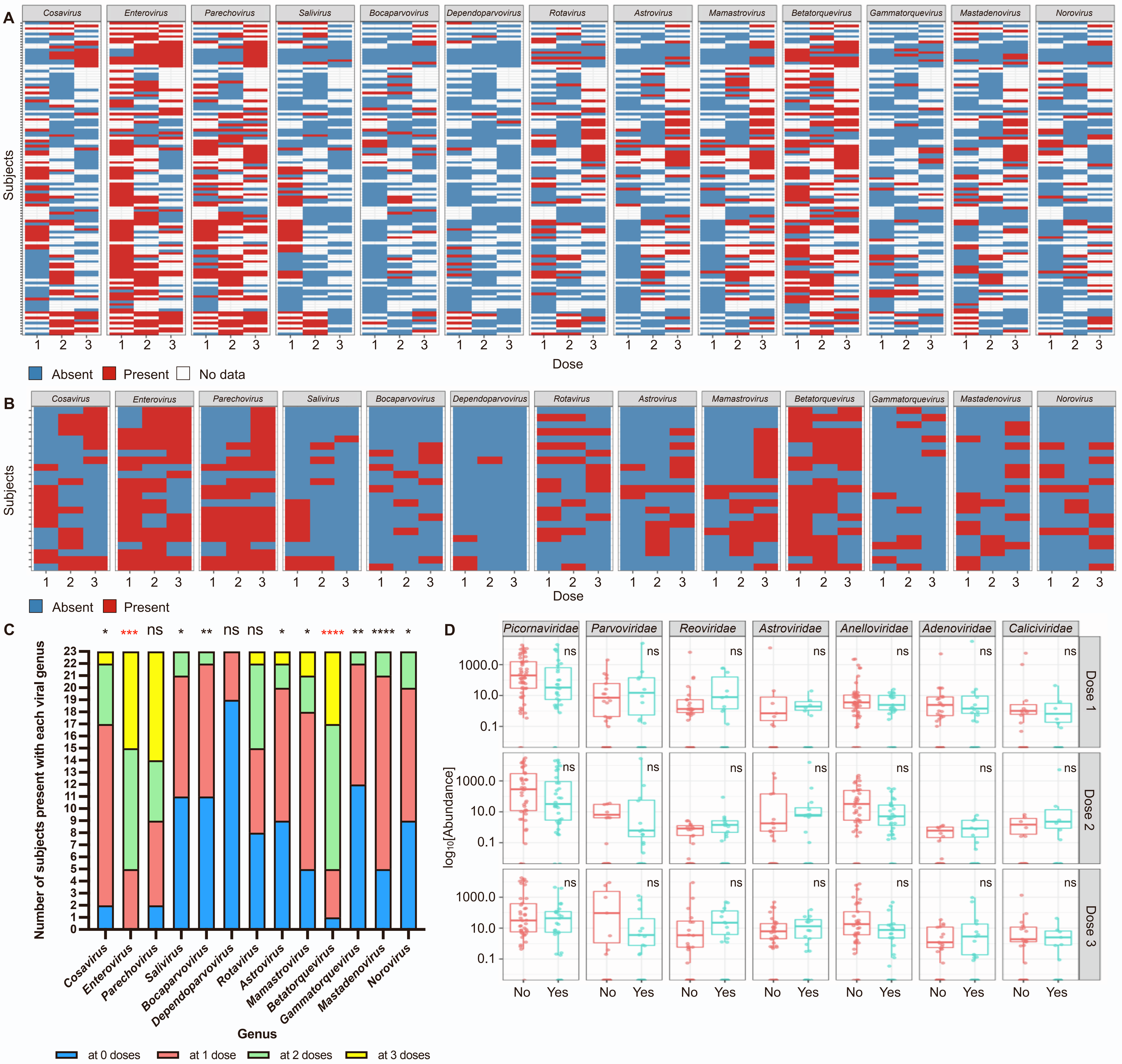

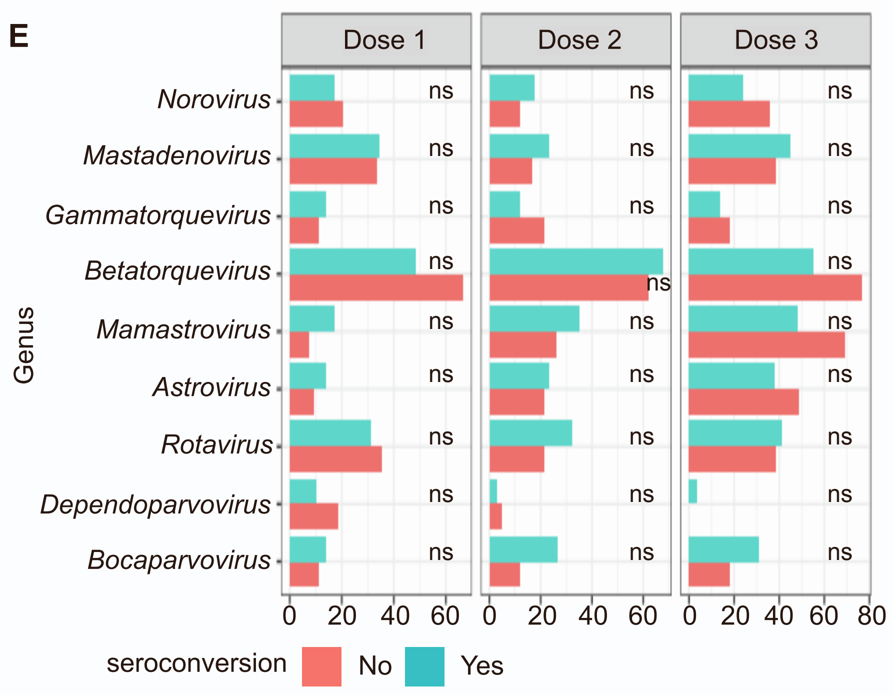

Figure S4. Viral persistence varies per viral family and seroconversion does not associate with mean abundance at the family level or percent positivity at the viral genera level, related to Figure 5. Presence and absence of each viral genus over time in **A)** all infants and **B)** a subset of 23 infants with data available at every dose. **C)** Number of infants with presence of each viral genus at one or more doses in the subset of 23 infants. Viral persistence per genus was defined as a significantly higher number of infants with presence of a viral genus at more than 1 dose as compared to at 1 dose alone and tested using Fisher's exact test (*p < 0.05, **p < 0.01, ***p < 0.001, ****p < 0.0001, ns = not significant). Red asterisk represents a significantly higher number of infants with virus present at greater than 1 dose compared to 1 dose alone and black asterisk represents a significantly higher number of infants for 1 dose alone compared to more than 1 dose. **D)** The log transformed mean abundance of eukaryotic viral families in Ghanaian infants identified as non-seroconverters ("No") and seroconverters ("Yes") at three doses (Dose 1, 2, 3). Statistical comparison between two serostatus groups was performed using Wilcoxon test (ns = not significant). n = 135 averaged non-seroconverter samples (Dose 1: 54, Dose 2: 42, Dose 3: 39) and 92 averaged seroconverter samples (Dose 1: 29, Dose 2: 34, Dose 3: 29). E) The presence (% of samples detected) of each viral genera with respect to serostatus.

Figure S5

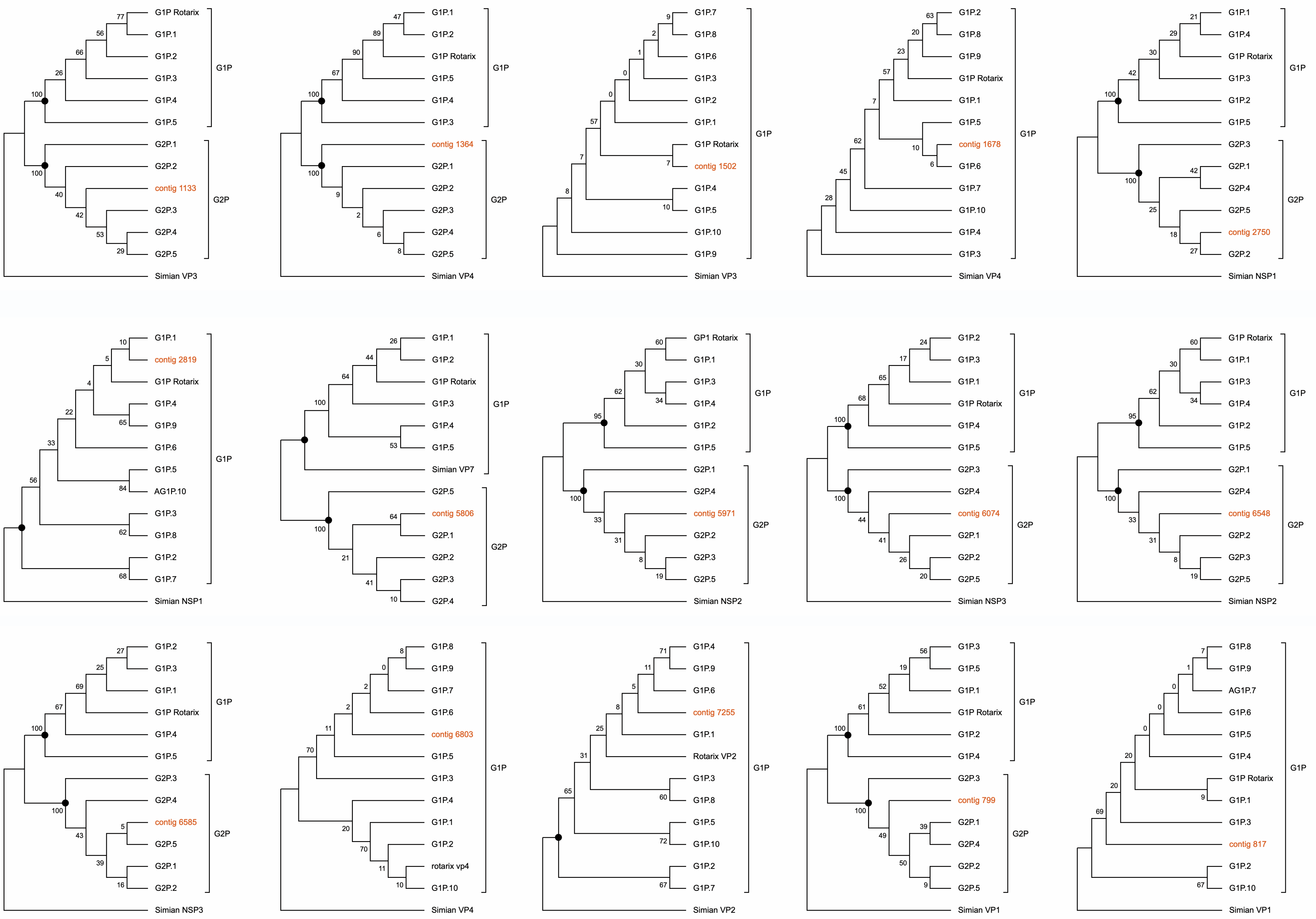

Figure S5. Strain Classification of rotavirus contigs, related to Figure 5. Phylogenetic analysis of rotavirus contigs (n = 15). Contigs were aligned to their top-10 hits resulting from a blastp search against NCBI nr. If both G1P and G2P were present in the top-10, then the top-5 G1P and G2P hits were collected for multiple sequence alignment. If only G1P or G2P were present, then the top 10 from that group was collected. Multiple sequence alignments to the top-10 were trimmed and the resulting alignment used for phylogenetic tree construction using maximum-likelihood inference (GTR+gamma). The Simian version of the aligned protein was used as an outgroup. Branch stability was determined using 100 bootstrap replicates. Metagenome assembled contig is indicated in orange. G1P and G2P brackets were applied when a branch was separated by a bootstrap > 95.

Figure S6

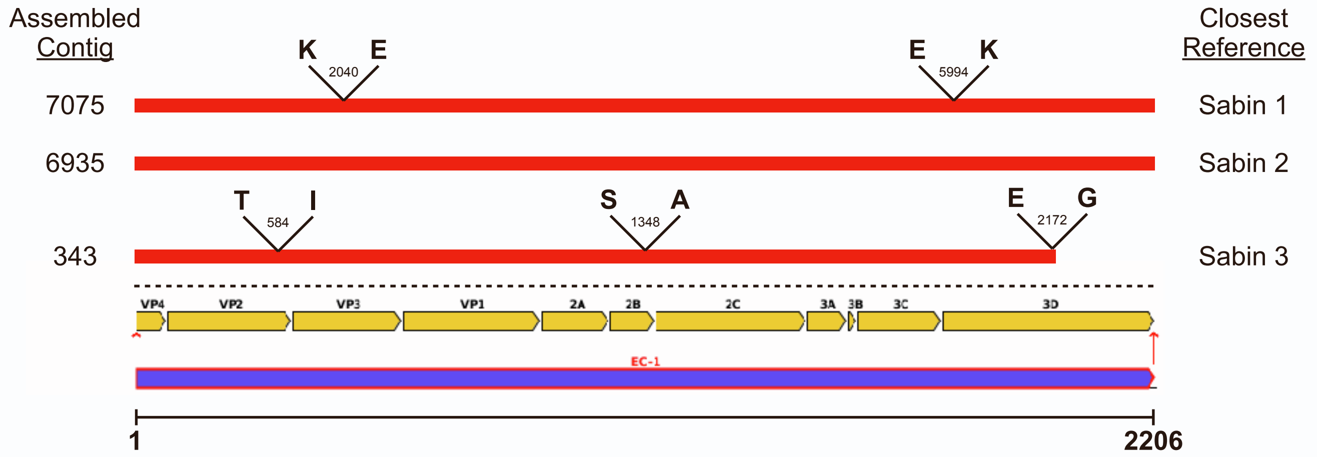

Figure S6. Assembled contig of oral polio vaccine strains, Sabin 1, Sabin 2 and Sabin 3, related to Figure 6. Assembly recovered a Sabin 1 contig containing a full-length polyprotein with two amino acid mutations (c.2040K>E in VP3 and c.5994E>K in RD3) when compared to published Sabin 1 genomes. The recovered Sabin 2 contig was full-length and identical to reference Sabin 2 polyprotein. The recovered Sabin 3 contig was 3' truncated by 32 amino acids of the 3D (RNA directed RNA polymerase) and had 3 mutations (c.584T>I in VP2, c.1348S>A in protein B and c.2172E>G in 3D).
