# Supplementary material for: Enteric virome negatively affects seroconversion following oral rotavirus vaccination in a longitudinally sampled cohort of Ghanaian infants
Source: Cell Host Microbe. 2022 Jan 12;30(1):110–123.e5. doi: 10.1016/j.chom.2021.12.002 (PMC8763403; doi:10.1016/j.chom.2021.12.002)
Supplement: Document S2. Article plus supplemental information [file mmc2.pdf]

Clinical and Translational Report
Cell Host & Microbe

Enteric virome negatively affects seroconversion following oral rotavirus vaccination in a longitudinally sampled cohort of Ghanaian infants

Graphical abstract

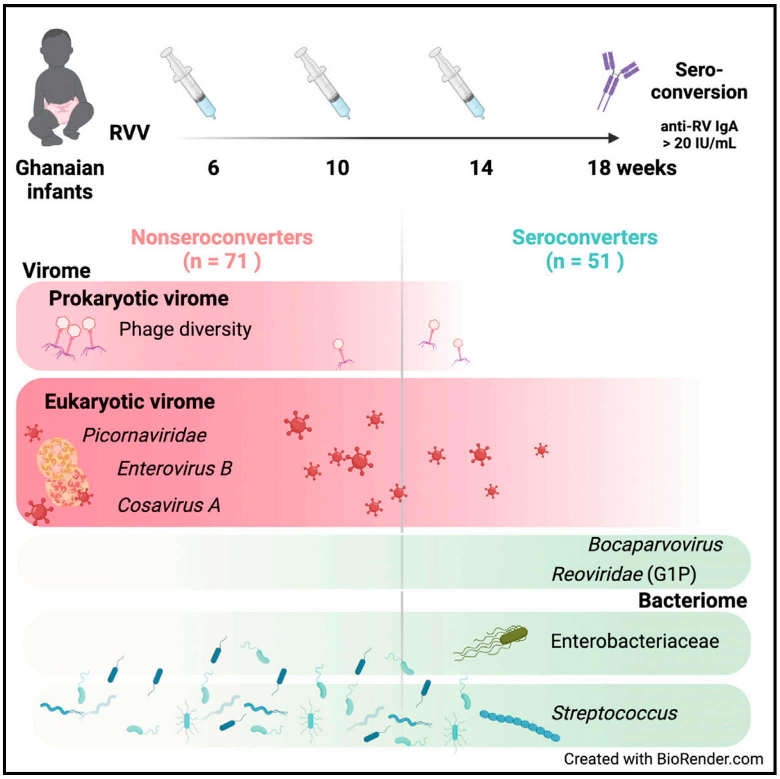

Authors

Andrew HyoungJin Kim, George Armah, Francis Dennis, ..., Megan T. Baldridge, Scott A. Handley, Vanessa C. Harris

Correspondence

v.c.harris@amsterdamumc.nl

In brief

Rotavirus vaccines have diminished performance in low- and middle-income countries, leaving children vulnerable to life-threatening gastroenteritis. Kim et al. employed unbiased metagenomic sequencing of longitudinally collected fecal samples from 122 Ghanaian infants and found that members of the virome were significantly associated with constrained rotavirus vaccine immunogenicity, potentially explaining this diminished performance.

Highlights

- Longitudinal analysis of microbiota of Ghanaian infants receiving rotavirus vaccine
- *Streptococcus* and Enterobacteriaceae taxa positively associate with RVV seroconversion
- *Enterovirus B*, *Cosavirus A*, and phage richness negatively associate with RVV serostatus

Kim et al., 2022, Cell Host & Microbe *30*, 110–123
January 12, 2022 © 2021 The Authors. Published by Elsevier Inc.
https://doi.org/10.1016/j.chom.2021.12.002

CellPress

CellPress
OPEN ACCESS

Cell Host & Microbe

Clinical and Translational Report

Enteric virome negatively affects seroconversion following oral rotavirus vaccination in a longitudinally sampled cohort of Ghanaian infants

Andrew HyoungJin Kim,[1,2] George Armah,[5] Francis Dennis,[5] Leran Wang,[2,3] Rachel Rodgers,[2,4] Lindsay Droit,[3] Megan T. Baldridge,[1,2,6] Scott A. Handley,[2,3] and Vanessa C. Harris[7,8,9,*]
[1]Division of Infectious Diseases, Department of Medicine, Washington University School of Medicine, St. Louis, MO, USA
[2]Edison Family Center for Genome Sciences & Systems Biology, Washington University School of Medicine, St. Louis, MO, USA
[3]Department of Pathology & Immunology, Washington University School of Medicine, St. Louis, MO, USA
[4]Department of Pediatrics, Washington University School of Medicine, St. Louis, MO, USA
[5]Noguchi Memorial Institute for Medical Research, College of Health Sciences, University of Ghana, Legon, Ghana
[6]Department of Molecular Microbiology, Washington University School of Medicine, St. Louis, MO, USA
[7]Department of Global Health (AIGHD), Amsterdam University Medical Center, Academic Medical Center, Amsterdam, the Netherlands
[8]Department of Medicine, Division of Infectious Diseases, Amsterdam University Medical Center, Academic Medical Center, Amsterdam, the Netherlands
[9]Lead contact
*Correspondence: v.c.harris@amsterdamumc.nl
https://doi.org/10.1016/j.chom.2021.12.002

SUMMARY

Rotavirus vaccines (RVVs) have substantially diminished mortality from severe rotavirus (RV) gastroenteritis but are significantly less effective in low- and middle-income countries (LMICs), limiting their life-saving potential. The etiology of RVV's diminished effectiveness remains incompletely understood, but the enteric microbiota has been implicated in modulating immunity to RVVs. Here, we analyze the enteric microbiota in a longitudinal cohort of 122 Ghanaian infants, evaluated over the course of 3 Rotarix vaccinations between 6 and 15 weeks of age, to assess whether bacterial and viral populations are distinct between non-seroconverted and seroconverted infants. We identify bacterial taxa including *Streptococcus* and a poorly classified taxon in Enterobacteriaceae as positively correlating with seroconversion. In contrast, both bacteriophage diversity and detection of *Enterovirus B* and multiple novel cosaviruses are negatively associated with RVV seroconversion. These findings suggest that virome-RVV interference is an underappreciated cause of poor vaccine performance in LMICs.

INTRODUCTION

Rotavirus (RV) is the leading cause of diarrheal mortality among children globally (GBD 2016 Causes of Death Collaborators, 2017; GBD 2016 Diarrhoeal Disease Collaborators, 2018). The burden of RV disease is borne disproportionately, with more than 90% of RV deaths in sub-Saharan Africa and southeast Asia (Tate et al., 2016; Troeger et al., 2018). Numerous live, attenuated oral rotavirus vaccines (RVVs) have been licensed for use since 2006, and their introduction in more than 100 countries has helped to substantially decrease RV deaths from more than 500,000 prior to vaccine introduction to approximately 120,000 deaths per year in children under five years of age (Li et al., 2021a; Troeger et al., 2018; ROTA Council, 2020). Despite this enormous public health accomplishment, the potential of RVVs has been limited by their diminished performance in low- and middle-income countries (LMICs). RVVs do not protect infants in high-income countries (HIC) and LMIC settings equally against severe RV gastroenteritis (RVGE) and death. In high-income countries, vaccine effectiveness is 84%–90%, while infants from LMICs only benefit from 45%–57% vaccine effectiveness (Jonesteller et al., 2017). This diminished performance helps explain why infants remain at risk for this life-threatening disease despite vaccine availability, as RV has remained the leading etiology of diarrheal hospitalizations and death in LMICs even after vaccine introduction (Jonesteller et al., 2017; Li et al., 2021a). In LMIC settings, improving RVV efficacy by even 15% could save an estimated 400,000 children's lives in the next two decades (Atherly et al., 2009; PATHCenters for Disease Control and Prevention CDC and World Health Organization WHO., 2006). This underscores the urgent public health need to understand and improve RVV performance in LMICs.

The etiology of the diminished performance of RVVs in LMICs remains elusive (Church et al., 2019a; Parker et al., 2018a). Numerous risk factors for vaccine failure, such as maternal antibodies, histo-blood group antigens, and malnutrition, have been identified and tested, but they fail to correspond with vaccine performance consistently across studies and geographic

This is an open access article under the CC BY license (http://creativecommons.org/licenses/by/4.0/).

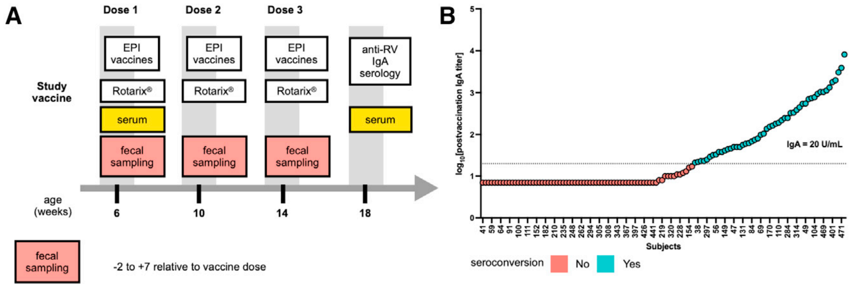

Figure 1. Study schematic

(A) 122 infants were administered Rotarix along with Expanded Programme on Immunization (EPI) vaccines: oral polio vaccine (OPV), pneumococcal conjugate vaccine (PCV), and pentavalent (diphtheria-pertussis-tetanus-haemophilus influenzae type b-hepatitis B) vaccine. Infants in this study also received OPVs and Bacillus Calmette-Guérin (BCG) at birth.

(B) Post-vaccination IgA titer from 122 subjects colored by serostatus.

settings (Church et al., 2019b; Grassly et al., 2016; Parker et al., 2018a, 2020; Praharaj et al., 2019). The intestinal microbiota is known to play a fundamental role in the development, education, and regulation of the host immune system and is also an important driver of inter-individual variation in immunity (Belkaid and Harrison, 2017; Neil and Cadwell, 2018). Mouse models have highlighted the importance of transkingdom bacterial-viral-immune interactions in the replication and control of enteric viruses (Baldridge et al., 2015; Erickson et al., 2018; Kuss et al., 2011; Pfeiffer and Virgin, 2016; Robinson et al., 2014; Stefan et al., 2020). Building on these findings, clinical studies have evaluated potential correlations between the bacterial microbiota and RVV immunogenicity in children in LMICs (Harris et al., 2018b; Kim et al., 2021). Previously, we reported that among infants in Ghana, the relative abundance of Bacteroidetes correlated negatively and bacteria related to *Streptococcus bovis* correlated positively with RVV immunogenicity (Harris et al., 2017). Yet, subsequent studies in India, Malawi, the UK, and Nicaragua have not replicated these findings, and few consistent correlations between bacterial microbiota and RVV immunogenicity have been found (Fix et al., 2020; Parker et al., 2018b, 2020; Sindhu et al., 2017).

Although the bacterial microbiota has been a major focus of research, infants host a remarkable diversity of eukaryotic viruses and bacteriophages (Holtz et al., 2014; Liang et al., 2020; Lim et al., 2015). These communities are dynamic over time, and children in LMICs have higher eukaryotic viral abundance and carriage when compared with infants in high-income settings (Gregory et al., 2020; Holtz et al., 2014; Reyes et al., 2015). Research to date has focused on testing whether specific enteric viruses, such as non-polio enteroviruses or oral polio vaccine, may impact RVV performance (Holtz et al., 2014; Parker et al., 2018b; Patel et al., 2012; Tan et al., 2020; Taniuchi et al., 2016). However, diverse communities of viruses inhabit the infant intestine and may also be considered as colonizers with the capacity to disrupt or promote immune development and homeostasis. By extension, these viral communities may alter immune responses to and replication of live oral attenuated RVVs (Dallari et al., 2021; Kernbauer et al., 2014; Neil and Cadwell, 2018). Unbiased evaluation of interactions between the infant virome and RVV performance is, therefore, warranted.

In this study, we aimed to evaluate the interactions of the bacterial microbiome and the eukaryotic and prokaryotic virome with host immunity to the Rotarix, an orally administered, live attenuated G1P8 RVV, in a retrospective, longitudinal cohort of infants from 6 to 15 weeks of age in Ghana. We employed unbiased metagenomic sequencing of the RNA and DNA virome in conjunction with 16S rRNA gene analysis of the bacterial microbiome. Our analysis reveals numerous constituents of the bacteriome and virome associated with seroconversion, thus identifying components of the microbiota that may influence development of effective RVV immune responses.

RESULTS

Study design and participants

This study retrospectively evaluated serially collected fecal samples from infants in Navrongo, a rural setting in northern Ghana, who were enrolled in a previously reported phase IV randomized clinical trial. The trial evaluated the immunogenicity of the Rotarix vaccine after different dosing schedules (NCT01575197, clinicaltrials.gov) (Armah et al., 2016). This nested study (Figure 1A), approved by the institutional review board of the Noguchi Memorial Institute for Medical Research in Ghana (#1492), included healthy infants enrolled in the three-dose Rotarix (at 6, 10, and 14 weeks) trial arm who were sero-negative for anti-RV IgA at baseline and who had at least one fecal sample available at 6, 10, or 14 weeks, with fecal samples collected 48 h prior to and 7 days following each vaccine dose. No significant difference was observed between 14 matched pre- and post-dose 1 vaccination samples in bacteriome composition (Figures S1A–S1C). Infants concomitantly received standard immunizations including the trivalent oral poliovirus vaccine (OPV) and pneumococcal conjugate and pentavalent vaccines. Serum samples were collected at 6 and 18 weeks of age. Seroconversion was defined as the detection of an anti-RV IgA concentration $\geq$ 20 U/mL at 18 weeks in infants who were seronegative (IgA < 20 U/mL) at the time of receipt of their first RVV (6 weeks) (Figures 1A and 1B) (Patel et al., 2013).

From the three-dose Rotarix trial arm of the original study, 122 (51 [42%] seroconverters and 71 [58%] non-seroconverters) of the 143 infants had fecal samples available and were included in the nested cohort. Baseline characteristics were similar between seroconverters and non-seroconverters (Table 1). From this cohort in which 53% of subjects (64/122) were females, 460 fecal samples were available, with 177 at dose 1 (6 weeks), 155 at dose 2 (10 weeks), 127 at dose 3 (14 weeks), and 1 which was not assigned to any dose

Different bacterial taxa are associated with seroconversion over a longitudinal timecourse

We performed sequencing-based analysis of the V4 region of the 16S rRNA gene for fecal samples obtained from both

Table 1. Characteristics of Ghanaian infant cohort

	Total subjects	Seroconverters	Non-seroconverters
Age (days)	43.09	43.02	43.14
Subjects, n (%)	122 (100%)	51 (42%)	71 (58%)
Female	64 (53%)	23 (36%)	41 (64%)
Nankam	40 (33%)	16 (40%)	24 (60%)
Kassem	80 (66%)	35 (44%)	45 (56%)
Other	2 (2%)	0 (0%)	2 (100%)
Malnutrition (z <−2), n (%)			
stunting (hfaz)	11 (9%)	4 (36%)	7 (64%)
wasting (wfhz)	5 (4%)	2 (40%)	3 (60%)
underweight (wfaz)	12 (10%)	3 (25%)	9 (75%)

Age indicated at the time of first vaccination (DS1). Nankam and Kassem are the predominant ethnicities in Navrongo, Ghana. Malnutrition indicated if height for age z-score (hfaz), weight-for-height z-score (wfhz), and/or weight-for-age z-score (wfaz) was less than −2.

non-seroconverters and seroconverters at doses 1, 2, and 3 of the vaccination time course. Analysis of the phylum-level composition demonstrated the presence of members of the phyla Actinobacteria, Bacteroidetes, Firmicutes, and Proteobacteria. There were no significant differences in the relative abundance of each bacterial phyla when comparing seroconverters to non-seroconverters (Figures 2A and S1D). Similarly, minimal changes in the relative abundance of each phyla were observed when examined over the course of the 3-dose regimen (Figure S1E). Consistent with these observations, no significant differences in alpha diversity (richness or Shannon diversity) over the longitudinal time course or beta diversity (weighted UniFrac distances) at any time point were observed between non-seroconverters and seroconverters (analysis of covariance [ANCOVA] and permutational multivariate analysis of variance [PERMANOVA], respectively, all $p > 0.05$; Figures 2B and 2C).

We performed DESeq2 analysis to identify taxa associated with seroconversion at each time point. We identified six amplicon sequence variants (ASVs) over the three time points that discriminated between non-seroconverters and seroconverters (Figure 2D; Table S1). The abundance of an unclassified taxon within the bacterial family Enterobacteriaceae (ASV 3) at dose 1 was associated with seroconversion. At dose 2, two taxa within the genus *Streptococcus* (ASV 4 and 5) as well as *Lactobacillus fermentum* (ASV 45) were associated with seroconversion, while another taxon in *Streptococcus* (ASV 6) was negatively associated. At dose 3, the same taxon in Enterobacteriaceae (ASV 3) again emerged as a biomarker for seroconversion, accompanied by *Rothia mucilaginosa* (ASV 32), an Actinobacteria (Figure 2D). We also performed multiple Pearson's correlation analyses between bacterial taxa and post-vaccination IgA titer. We identified 1 ASV significantly correlated with post-vaccination IgA titer (Figure 2D). Specifically, at dose 3, *Eggerthella lenta* was positively correlated with post-vaccination IgA titer (Pearson's rho = 0.453, p value = 0.0484). These data indicate that while the overall composition of the bacterial microbiota over the time course of vaccination does not vary between non-seroconverters and seroconverters, specific taxa belonging to the orders Enterobacteriales and Lactobacillales and the Actinobacteria phylum are consistently or periodically associated with seroconversion.

Phageome richness and diversity is negatively associated with seroconversion and decreases over time

We next explored whether prokaryotic viruses (bacteriophage) may be implicated in RVV seroconversion by analyzing the phageome in our cohort. Shotgun sequencing was performed on both DNA and RNA isolated from virus-like particle (VLP) preparations from the longitudinally collected fecal samples. Per-sample abundances were determined for all contigs longer than 1,000 bases that were assigned to a phage taxonomic lineage (Figure S2). We found that the phageome was dominated by phages from the order *Caudovirales*, represented by the families *Ackermannviridae*, *Myoviridae*, *Podoviridae*, and *Siphoviridae* (Figure 3A). *Microviridae* were only detected in 6 samples (4 samples at dose 2 and 2 samples at dose 3) and were, therefore, excluded from additional analysis. Analysis of the relative abundances of the *Caudovirales* families did not reveal any differences specific to seroconversion (Wilcoxon signed-rank test, $p > 0.05$; Figure S3A). Similarly, the presence or absence of any one family of phage was not associated with seroconverter status (Fisher's exact test, all $p > 0.05$; Figure S3B). Interestingly, *Podoviridae* were the most prevalent phages identified in samples at doses 1 and 2, but by dose 3, *Siphoviridiae* overtook the community (Figure S3C). Similarly, *Ackermannviridae* were only detected at doses 1 and 2 but were absent by dose 3, indicating a transformation in the enteric phageome between 6 and 15 weeks of life in Ghanian infants (Figure S3C).

Phage richness and Shannon diversity decreased, on average, in all samples over time (ANOVA, richness: $p = 2.98 \times 10^{-06}$, Shannon diversity: $p = 1.04 \times 10^{-5}$; Figure 3B). Samples from non-seroconverters had significantly increased richness and Shannon diversity, most strikingly at dose 1, when compared with seroconverters (ANCOVA, richness: $p = 0.004$, Shannon diversity: $p = 0.006$; Figure 3B). Similarly, beta diversity (Bray-Curtis dissimilarity, non-metric multi-dimensional scaling [NMDS]) was also significantly different between seroconverters and non-seroconverters at dose 1 (PERMANOVA, $p = 0.049$, permutations = 1×10^5; Figure 3C). However, this p value ranged from 0.063 to 0.046 over 10 permutations, suggesting that phageome beta diversity is only marginally different between seroconverters and non-seroconverters. PERMANOVA analysis at doses 2 and 3 consistently resulted in $p > 0.5$, suggesting that the phageome community composition was similar between seroconverters and non-seroconverters between 10 and 14 weeks of life.

We compared phage richness and Shannon diversity with bacterial richness and Shannon diversity and found that at dose 1, phage Shannon diversity was positively correlated to bacterial Shannon diversity when examining all samples (Spearman rho = 0.302, $p = 0.007$) and for non-seroconverters (Spearman rho = 0.424, $p = 0.002$; Figure 4D). These correlations were not significant at doses 2 or 3 ($p > 0.05$; Figures 4E and 4F). Phage richness (ANCOVA, $p = 0.029$) and Shannon diversity (ANCOVA, $p = 0.020$) were higher in samples from non-seroconverters than in seroconverters at dose 1 only (Figure 4D). Taken

CellPress
OPEN ACCESS

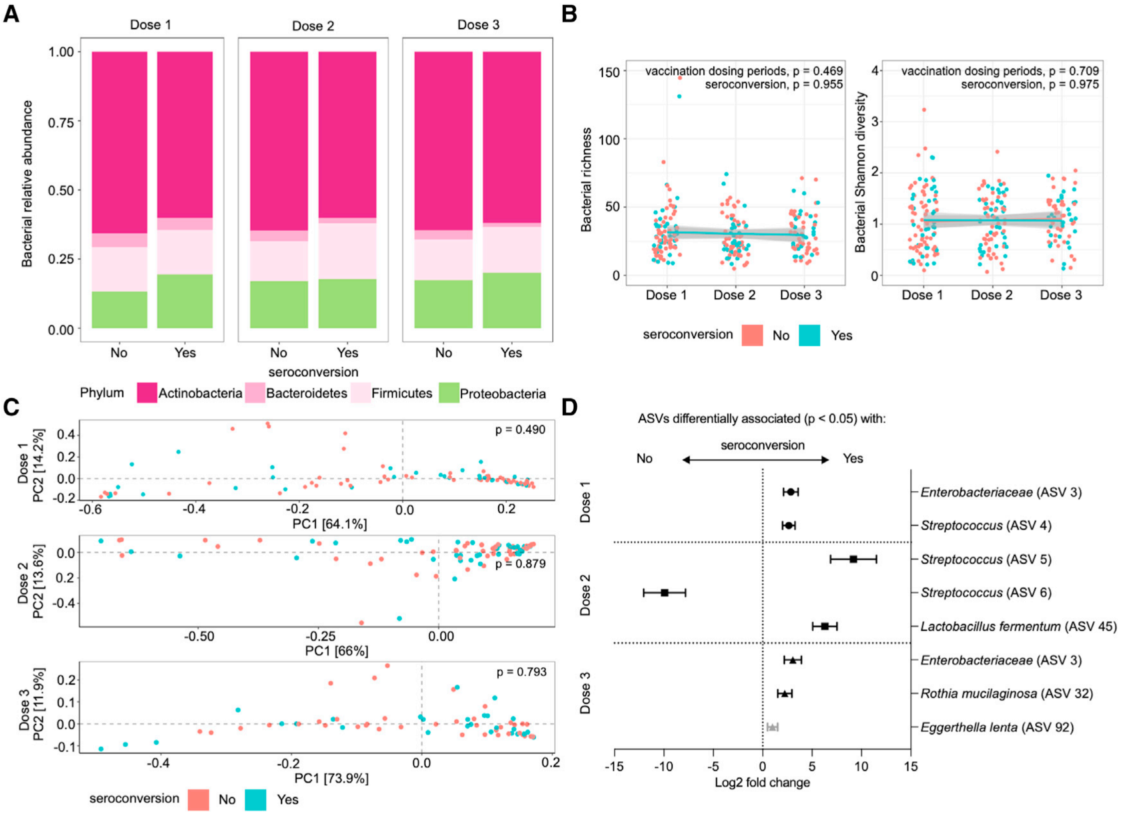

Figure 2. Specific bacterial taxa are associated with serostatus over a longitudinal time course

(A) Average proportions of each bacterial phylum in the total bacteriome composition for Ghanaian infants identified as non-seroconverters (“no”) or seroconverters (“yes”) over three doses (doses 1, 2, and 3).

(B) Bacterial richness and diversity at each dosing period with linear models showing correlation with each serostatus across dosing periods. p values for across dosing period and serostatus comparisons were calculated using one-way ANOVA and ANCOVA tests, respectively. Lines depict the linear model while greyed areas indicate the 95% confidence level interval of the model for each group.

(C) Bacterial beta diversity (weighted UniFrac distance) of samples at doses 1, 2, and 3. Wilcoxon tests and permutational multivariate analysis of variance (ADONIS) were used to compare between serostatus groups in alpha and beta diversity analyses, respectively.

(D) Summary plot showing bacterial ASVs identified using DESeq2 or multiple Pearson’s correlation analyses between abundance of all bacterial ASVs at both genus and species level and postvaccination IgA titers at each dosing period. Log fold change (symbols; dose 1: ●, dose 2: ■, dose 3: ▲) and log fold change standard error of the mean (line) are indicated. Black symbols indicate markers selected from DESeq2 analyses, and gray symbol indicates markers selected from multiple Pearson’s correlation analysis. Wald test was used to compare groups and Pearson’s correlation coefficient analysis was used for multiple Pearson’s correlation analyses. n = 148 averaged non-seroconverter samples (dose 1: 60, dose 2: 49, dose 3: 39) and 99 averaged seroconverter samples (dose 1: 35, dose 2: 35, dose 3: 29).

together, increases in phage alpha diversity (both richness and Shannon diversity), marginal differences in beta diversity, and differential relationships to bacterial alpha diversity indicate a unique phageome at dose 1 associated with a lack of seroconversion to the RVV.

Virome analysis reveals temporal shifts in eukaryotic viruses across age

We next evaluated the eukaryotic virome. Evidence for the presence of seven dominant viral families (*Picornaviridae*, *Parvoviridae*, *Reoviridae*, *Astroviridae*, *Anelloviridae*, *Adenoviridae*, and *Caliciviridae*), which include both single- and double-stranded RNA and DNA viruses, was found across the cohort (Figures 5A–5C). Of these, reads assigned to the family *Picornaviridae* were the most abundant (Figure 5A). With the exception of reads assigned to the family *Anelloviridae*, the majority of reads were assigned to each viral family with greater than 70% identity to a sequence in the reference database (quadrant 1 [Q1]: 56.2%, Q2: 35.0%) (Figure 5B). Most *Anelloviridae* reads were assigned with lower (< 70%) identity (Q3: 40.6%, Q4: 40.8%), with alignment statistics suggesting that *Anelloviridae* detected in this cohort were likely novel (low % ID to a sequence in the reference database over long lengths), while those from the other six families were similar to viral sequences previously deposited into reference databases. The average number of viral genera detected per infant at each dose were 6.23 at dose 1, 5.97 at dose 2, and 6.87 at dose 3. There was no significant difference in the number of viral taxa detected between seroconverters and non-seroconverters at any dose (Fisher’s exact test, p = 1 at all doses).

Analysis of each viral family over time indicated that reads assigned to *Reoviridae*, which include RVs (p = 0.022 [dose 2

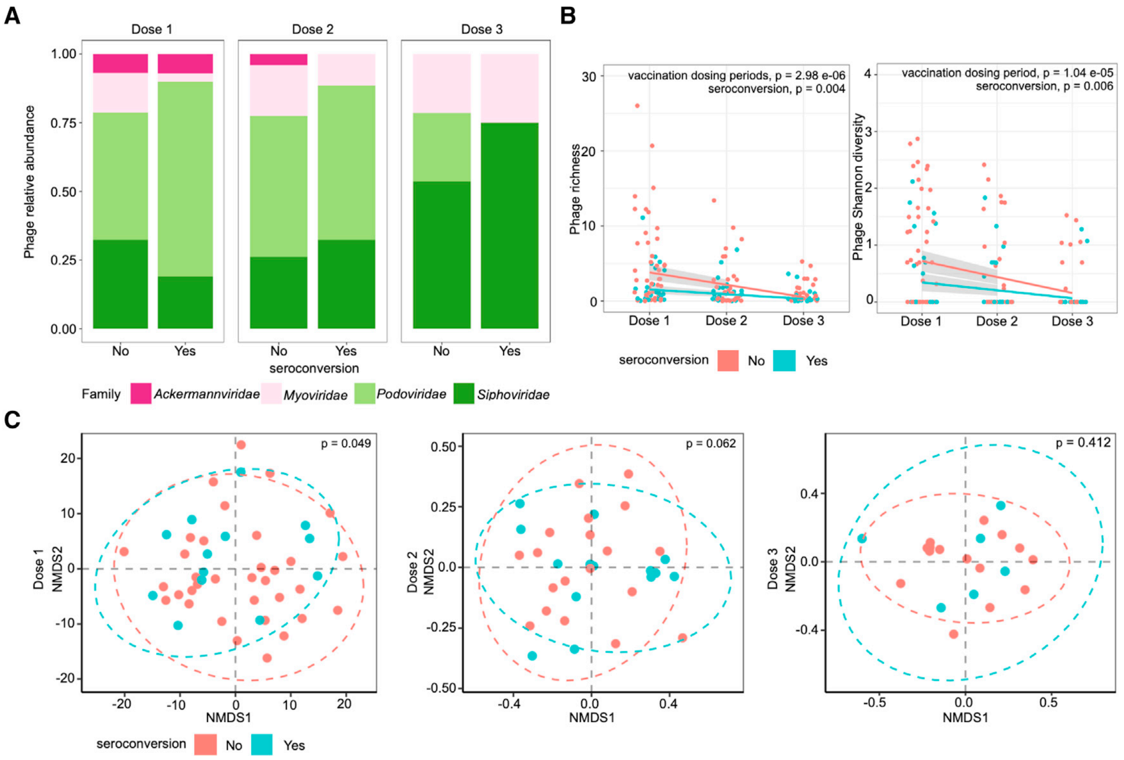

Figure 3. Phage alpha diversity at dose 1 is negatively associated with seroconversion

(A) Average proportions of each phage family in the total phageome composition for Ghanaian infants identified as non-seroconverters ("no") or seroconverters ("yes") over three doses (doses 1, 2, and 3).

(B) Phage richness and diversity at each dosing period with linear models showing correlation with each serostatus across dosing periods. p values for across dosing period and serostatus comparisons were calculated using one-way ANOVA and ANCOVA tests, respectively. Lines depict the linear model while greyed areas indicate the 95% confidence level interval of the model for each group.

(C) Phage beta diversity (non-metric multi-dimensional scaling [NMDS]) of samples at dose 1, dose 2, and dose 3. Statistical differences of beta diversity and alpha diversity between serostatus groups were evaluated using and the Wilcoxon test and permutational multivariate analysis of variance (ADONIS), respectively. n = 216 averaged non-seroconverter samples (dose 1: 54, dose 2: 84, dose 3: 78) and 162 averaged seroconverter samples (dose 1: 30, dose 2: 72, dose 3: 60).

to dose 3]) and *Astroviridae* ($p = 4.34 \times 10^{-5}$ [dose 1 to dose 3] and p = 0.015 [dose 2 to dose 3]) increased in abundance over time, while the abundance of reads assigned to other viral families remained the same (Figure 5C). We also assessed whether changes in the abundance over viral genera over time might be due to novel infections or persistence of infections within individual infants. The analysis was constrained by the number of infants with samples available at every dose (Figures S4A and S4B); however, *Enterovirus* and *Betatorquevirus* had high persistence with higher numbers of infants with viral reads present across doses 2 and 3, as compared with dose 1 alone (Fisher's exact test, $p > 0.05$; Figure S4C).

We assessed whether mean abundances or the presence (percentage of samples detected) of each viral family was associated with RVV seroconversion and found no significant differences at the viral family level between non-seroconverters and seroconverters at all 3 doses (Wilcoxon signed-rank test, $p > 0.05$, Fisher's exact test, $p > 0.05$; Figures S4D and S4E).

To assess if the RVV itself altered viral composition, seven paired pre- and post-vaccine samples were analyzed for the prevalence of viral families (*Picornaviridae*, *Reoviridae*, *Astroviridae*, *Anelloviridae*, *Adenoviridae*, and *Caliciviridae*) (Figure S4D). The prevalence of each viral family was similar in both pre- and post-vaccine samples (Fisher's exact test adjusted $p > 0.05$). However, due to the sampling deficit in pre-vaccine samples (n = 7, 8.4% of total samples), this analysis is a minimal representation of the total patient population and should be interpreted accordingly.

Non-vaccine strain *Reoviridae* increases over time

In order to determine if all of the reads assigned to the *Reoviridae* were originating from the Rotarix vaccine or from other *Reoviridae*, we examined assembled contigs to obtain more genetic information than that were available from individual reads. Fifteen *Reoviridae* contigs were identified, all of which were assigned to different protein coding segments of rotavirus genomes (Figure S5). Contigs with sequence similar to 8 different segments of the rotavirus genome were identified (NSP1-3 and VP1-4 and VP7) (Figure S5). Phylogenetic analysis revealed that each contig could be placed into either a G1P or G2P clade. The Rotarix vaccine strain is in the G1P clade, so those placed in the G2P clade are likely non-vaccine naturally circulating RV strains. Non-vaccine strains in the G1P clade cannot be ruled out. Due to the random region of each

CellPress
OPEN ACCESS

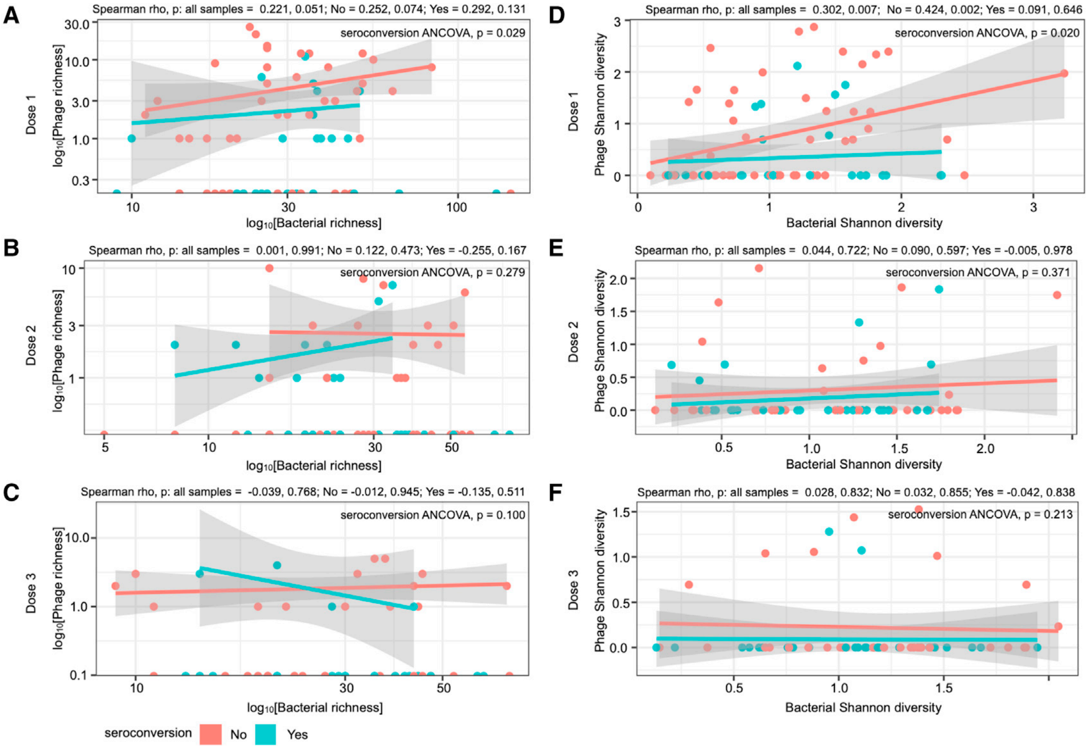

Figure 4. Phage diversity significantly correlated with bacterial diversity at dose 1

The correlation analysis of phage and bacterial (A–C) richness and (D–E) Shannon diversity at three doses (doses 1, 2, and 3) using analyses of Spearman's rank-order correlation (Spearman rho) and covariance (ANCOVA). Lines depict the linear model while grayed areas indicated the standard error of the mean for each group.

gene segment obtained via metagenomic assembly, it was not possible to determine if substrains of G1P or G2P were present in each sample. When examining the prevalence of G1P or G2P strains over time, there was a clear emergence of G2P strains at doses 2 and 3 with the majority being identified in dose 3 samples; however, there was no significant difference between seroconversion status at any dose (Figure 5E). We compared the prevalence of G1P or G2P strains between doses. G1P strain detection was significantly decreased in dose 2 compared with dose 1 (Fisher's exact test, p = 0.038) and increased in dose 3 compared with dose 2 (Fisher's exact test, p = 0.045) only in non-seroconverters. G2P strain detection was significantly increased in dose 3 compared with dose 1 or dose 2 in both non-seroconverters (Fisher's exact test, p = 0.01 [dose 1 to dose 3] and p = 0.004 [dose 2 to dose 3]) and seroconverters (Fisher's exact test, p = 0.011 [dose 1 to dose 3] and p = 0.019 [dose 2 to dose 3]; Figure 5F).

Enterovirus B and novel *Cosavirus A* are associated with a lack of seroconversion

Because enteroviruses and OPV strains have previously been implicated in limiting RVV efficacy, we closely examined the *Picornaviridae* family in our cohort (Figure 6) (Parker et al., 2018b; Patel et al., 2012; Taniuchi et al., 2016). Reads were primarily assigned to four *Picornaviridae* genera: *Enterovirus*, *Parechovirus*, *Salivirus*, and *Cosavirus* (Figure 6A). We separated reads assigned to *Enterovirus C* and *Enterovirus B*, as *Enterovirus C* reads are likely to originate from OPV strains (Sabin 1, 2, and 3). In addition, small numbers of reads were also assigned to other *Picornaviridae* including the genera *Sapelovirus* (n = 52), *Cardiovirus* (n = 45), and *Kobuvirus* (n = 26) but were excluded from further analysis due to their overall rarity (0.002%–0.001% of analyzed reads). While reads summed at the family level of *Picornaviridae* exhibited no change over time (Figure 5C), when split into individual genera, the abundance of reads assigned to *Enterovirus*, including both *Enterovirus C* (p = 0.026 [dose 2 to dose 3]; p = 5.6×10^{-4} [dose 1 to dose 3]) and *Enterovirus B* (p = 4.79×10^{-3} [dose 1 to dose 3]) as well as *Salivirus* (p = 3.99×10^{-3} [dose 1 to dose 2]; p = 1.25×10^{-3} [dose 2 to dose 3]; p = 1.04×10^{-9} [dose 1 to dose 3]), decreased over time (Figure 6A). In contrast, reads assigned to *Parechovirus* and *Cosavirus* were consistently abundant over time (p > 0.05), indicating independent longitudinal trajectories for individual *Picornaviridae* genera during early life development.

We next explored the relationships between the detection (presence or absence) of *Picornaviridae* species in the stool with RVV seroconversion. We observed that at dose 1, the detection of *Enterovirus B* (Fisher's exact test, p = 0.002) or *Cosavirus A* (p = 0. 039) was more frequent in samples from subjects that did not seroconvert (Figure 6B). *Parechovirus A* was detected less frequently in seroconverters but only at dose 3 (Fisher's exact test, p = 0.02; Figure 6B).

Contigs from the 3 *Enterovirus C* strains (Sabin 1, 2, and 3) used in the OPV were identified and quantified using per-sample

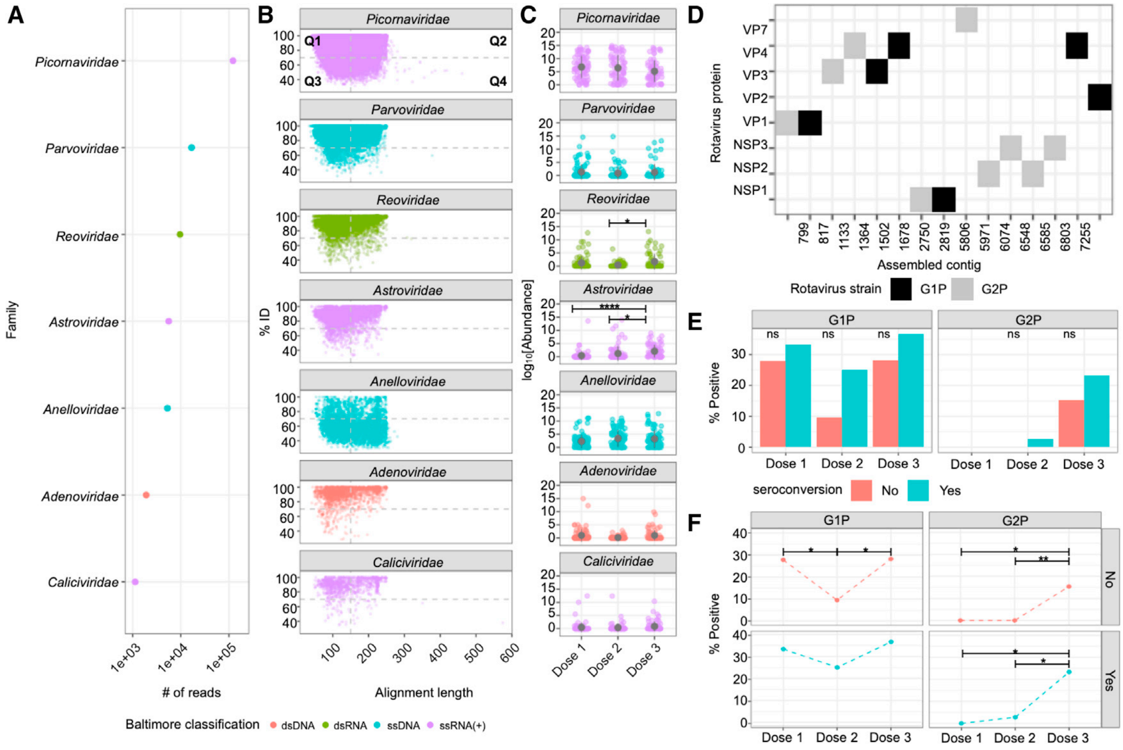

Figure 5. Infant eukaryotic enteric viruses. Analysis of DNA and RNA virus sequences obtained from VLP sequencing

(A) The total number of reads assigned to each eukaryotic viral family from all samples (n = 316).

(B) Scatterplot showing alignment statistics of reads assigned to viral families, with each point representing a single metagenomic sequence plotted in relation to the percent identity to the reference database sequence (y axis) and the reference alignment length (x axis). Alignment lengths longer than 250 are due to reference alignments with interspersed insertions relative to the query. A dashed line is provided at 70% ID on the y axis and 150 bases on the x axis as a guide for high/low identity and short/long alignment lengths. This breaks each plot into four reference quadrants (Q1, Q2, Q3, and Q4).

(C) Viral family abundance at each vaccine dosing period, with mean and standard error of the mean (SEM) depicted. Points are colored based on Baltimore classification in (A–C).

(D) Rotavirus proteins encoded in assembled rotavirus contigs and their G1P/G2P assigned taxonomy.

Prevalence of each rotavirus contig at each dose comparing between (E) seroconversion status and (F) doses. Means are compared using the Kruskal-Wallis test followed with Dunn's post-hoc test. Adjusted p values are indicated with an asterisk. Prevalence is compared using Fisher's exact test followed by Bonferroni test (*p < 0.05, **p < 0.01, ***p < 0.001, ****p < 0.0001, ns = not significant).

read mapping (Figures 6C and 6D). These contigs were truncated at both 5′ and 3′ ends, but we were able to recover complete or near-complete sequences of the *Enterovirus C* polyprotein gene (Figure S6). Assembly recovered a Sabin 1 contig containing a full-length polyprotein with two amino acid mutations (c.2040K>E in VP3 and c.5994E>K in RD3) when compared with published Sabin 1 genomes. The recovered Sabin 2 contig was full length and identical to the reference Sabin 2 polyprotein. The recovered Sabin 3 contig was 3′ truncated by 32 amino acids of the 3D (RNA-directed RNA polymerase) and had 3 mutations (c.584T>I in VP2, c.1348S>A in protein B, and c.2172E>G in 3D). Abundances of these OPV contigs decreased over time and at similar rates in both seroconverters and non-seroconverters with 71.5% of samples having one or more strains at dose 1 compared with 59.0% at dose 2 and 34.8% at dose 3 (Fisher's exact test, dose 1, p = 1.000; dose 2, p = 0.647; dose 3, p = 0.611; Figures 6C and 6D).

Metagenomic assembly recovered 5 *Cosavirus* contigs with relatively low percent identity to reference database cosaviruses (Figure 6D). Phylogenetic analysis of the full-length polyprotein open reading frame revealed that 4 contigs (320, 337, 328, and 339) make up a sub-clade to *Cosavirus A*. While one contig (336) was closely related to *Cosavirus D,* it was still distinct (Figure 6E). Overall, these data suggest that the presence of "competing" enteroviruses early in the vaccination time course may negatively affect RVV seroconversion and highlight a potentially important role for the understudied enterovirus *Cosavirus*.

Parvoviridae and *Reoviridae* abundance associate with higher post-vaccination IgA titer

Lastly, we performed multiple Pearson's correlation analyses between the abundance of detected viral taxa and post-vaccination IgA titers. We observed significant correlations between IgA and *Parvoviridae* (Pearson's rho = 0.426, p = 0.0433) and *Reoviridae* (Pearson's rho = 0.420, p = 0.0433) at dose 1 (Figure 6F). *Parvoviridae* genus *Bocaparvovirus* (Pearson's rho = 0.431, p = 0.0000955), but not *Dependoparvovirus*, was positively correlated with post-vaccination IgA titers (Figure 6G). The *Reoviridae* at dose 1 is exclusively reflective of vaccine strain G1P (Figure 5F). These data suggest that higher levels of RVV as well

CellPress
OPEN ACCESS

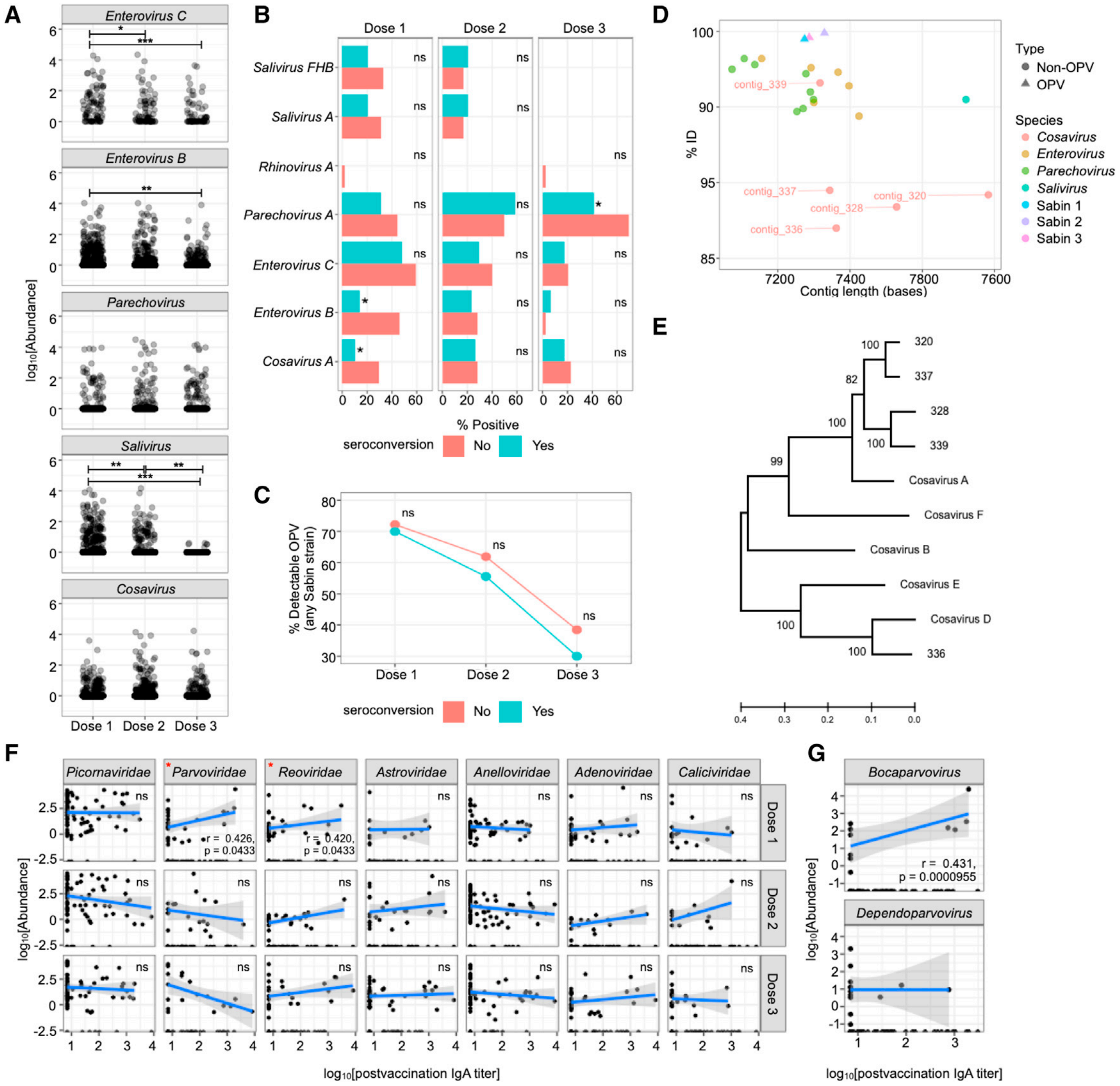

Figure 6. *Enterovirus B* and *Cosavirus A* are negatively associated with seroconversion

(A) Abundance of each genera read within the *Picornaviridae* family at each vaccine dosing period.

(B) The percentage of samples positive for each species of *Picornaviridae* at each dosing period.

(C) The percentage of samples positive for OPV, specifically Sabin 1, Sabin 2, and Sabin 3, at each dosing period. Means are compared using the Kruskal-Wallis test followed with Dunn's post-hoc test. Adjusted p values are indicated with an asterisk. Prevalence is compared using Fisher's exact test followed by Bonferroni test (*p < 0.05, **p < 0.01, ***p < 0.001, ****p < 0.0001, ns = not significant)

(D) Percent identity and assembled contig length of Pircornavirus species.

(E) Phylogenetic comparison of novel cosavirus sequences identified with reference cosaviruses. The tree was computed using trimmed, full-length aligned polyprotein sequences using maximum likelihood inference (General Time Reversible [GTR] + gamma distribution) and branch stability assessed using 100 bootstrap replicates.

(F) Correlation between viral taxa at family level and post-vaccination IgA titers.

(G) Correlation between two genera belonging to *Parvoviridae* and post-vaccination IgA titer. Adjusted p values were attained for each viral taxa by performing Pearson's correlation analysis followed by correction using the Bonferroni method. Lines depict the linear model while greyed areas indicate the 95% confidence level interval of the model.

as *Bocaparvovirus* in early phases of vaccine administration may enhance RVV responses.

DISCUSSION

RV remains a major killer of young children in LMICs, and understanding the determinants of RVVs' diminished performance in these settings is a global health priority. We describe the interactions of the bacterial and viral fecal microbiome with RVV immunogenicity in a longitudinal cohort of 6- to 15-week-old infants in rural Ghana. We found that numerous components of the human microbiota, including specific bacterial taxa, bacteriophage diversity, and known and novel eukaryotic viruses, correlate significantly with RVV immunogenicity in early life,

underscoring the importance of the microbiota in determining early human immune responses to enteric pathogens and oral vaccines.

A growing body of literature has evaluated correlations between the fecal bacterial microbiota and RVV immunogenicity, with results varying substantially by study and geographic settings. Here, we show that while bacterial diversity indexes do not correlate with RVV immunogenicity, specific taxa are associated with RVV immunogenicity over time in rural Ghanaian infants. These include a poorly classified taxon in *Enterobacteriaceae* at doses 1 and 3 and two taxa within the *Streptococcus* genus at dose 2. There is some overlap in these taxonomic findings with our previous work evaluating a parallel cohort of Ghanaian infants (from the same phase IV trial), wherein the bacterial microbiome was evaluated using chip technology rather than sequencing (Harris et al., 2017). In this parallel cohort, bacteria belonging to the *Streptococcus bovis* group correlated positively with seroconversion, whereas several Bacteroidetes were negatively correlated. We have also identified positive correlations between *Enterobacteriaceae* and RVV immunogenicity in a small urban cohort of infants in Karachi, Pakistan (Harris et al., 2018a). Interestingly, in a large well-conducted study evaluating determinants of RVV immunogenicity in the UK, Malawi, and India, *Escherichia* (*Enterobacteriaceae*) and *Streptococcus* were the two microbiota variables that were the most predictive of anti-RV IgA concentration among RV-naive infants in India (Parker et al., 2020).

Other microbiota correlations with RVV immunogenicity have been less reliably recapitulated across geographic settings;thus, the broad applicability of our study may be limited by our use of a single location. In aggregate, our findings suggest that selected bacterial taxa correlate with increased RVV immunogenicity in settings like Ghana; however, if they are causal to RVV immunity, their effect size may be limited or context-specific to this geographic setting. Further, these positive bacterial correlations are insufficient to explain the poor performance of RVVs in LMIC settings which, as our data suggest, may be better explained by viral-viral interference.

Our study represents the first unbiased, metagenomics-based exploration of the role of the virome in RVV immunogenicity. Interestingly, we found that increased phage alpha diversity at the time of the first dose of RVV correlated with poor RVV seroconversion. Bacteriophages can be both temperate, integrating into the bacterial chromosome or lytic, infecting and exploiting bacterial machinery to produce more virions to be released by host cell lysis (Federici et al., 2021). Bacteriophage-bacterial microbiome interactions can therefore have both predator-prey reciprocal and linear relationships (Lim et al., 2015). Other studies in early childhood suggest that bacteriophages accompany colonizing bacteria in early months of life followed by a contraction over time and may progress in a stepwise fashion with eukaryotic viruses (Liang et al., 2020; Lim et al., 2015). We found that phage diversity decreases only in non-seroconverters over this narrow window of 6 to 15 weeks of age, with no corresponding alterations in bacterial diversity. This finding raises two hypotheses for the role of bacteriophages in determining RVV immunogenicity. First, phages may alter RVV seroconversion through their interactions with specific bacterial taxa. Alternatively, phages may interact with RVV immune responses independently of the bacterial microbiota. Some phages have been shown to increase host antiviral cytokines, such as IFN-γ, independent of bacteria (Gogokhia et al., 2019), and expanded phage populations have also been linked to inflammatory enteric diseases such as Crohn's disease (Gogokhia et al., 2019; Norman et al., 2015). This capacity for phage-specific effects on the host suggests that phages could modulate host immunity to RVV in a manner independent of their bacterial hosts (Federici et al., 2021).

Despite its likely impacts on early host immunity, the eukaryotic virome has been insufficiently characterized in young infants, particularly in LMICs (Gregory et al., 2020). Factors determining the composition of the infant enteric eukaryotic virome in LMICs are poorly understood, but environmental exposures are likely important and include administration of oral viral vaccines (RVV/OPV), breastfeeding (Liang et al., 2020; Parker et al., 2020), seasonality, and living conditions, particularly animal exposures, sanitation, and water quality (Altan et al., 2018; Gregory et al., 2020; Liang and Bushman, 2021; Lim et al., 2015, 2016; Maqsood et al., 2019; Tan et al., 2020). Our findings suggest that LMIC infants may have earlier and more frequent eukaryotic infections than HIC infants. Among the 6- to 15-week-old infants in our cohort, the eukaryotic virome was extraordinarily rich and diverse. This is in line with research suggesting higher fecal carriage of eukaryotic viruses in LMIC as compared with high-income settings. These Ghanaian infants may also have earlier prominence of eukaryotic viral infections than western infants, as eukaryotic viruses become prominent in western cohorts around 4 months of life (Gregory et al., 2020; Holtz et al., 2014; Liang et al., 2020; Rampelli et al., 2017). Virome comparisons across populations and ages are limited by differences in study methodologies, including extraction techniques, frequent omission of analysis of RNA viromes, and lack of paired, longitudinal sampling strategies (Wang, 2020).

Picornaviridae dominated the virome of these Ghanaian infants, a finding consistent with a limited number of other studies characterizing the virome in similarly aged LMIC infants (Altan et al., 2018; Liang et al., 2020; Mogotsi et al., 2020; Tan et al., 2020). Viral family abundance in these infants was dynamic, even over a 9-week time frame. Select *Picornaviridae* family abundance, namely enteroviruses and saliviruses, contracted between 6 and 15 weeks of life. Enterovirus decreases were driven in part by decreased shedding of exogenously administered OPV strains across vaccination doses. In contrast, astroviruses and reoviruses increased over time.

As expected, because infants received Rotarix, many exhibited detectable levels of the G1P strains over time. Dose 1 G1P *Reoviridae* shedding also correlated significantly with anti-RV IgA titers, in line with previous literature (Cowley et al., 2017; Taniuchi et al., 2016). However, the total *Reoviridae* family shedding increased significantly at dose 3, primarily due to increased detection of G2P or natural circulating RV. This suggests that RV vaccination protects poorly against asymptomatic natural G2P RVs, even as early as 4 weeks after vaccination. While there were no differences in G1P and G2P shedding over time and seroconversion, it is possible that naturally circulating non-vaccine rotaviruses are important confounders of post-vaccination anti-RV IgA, complicating the use of this

readout as a correlate of RVV protection. Similar findings were recently shown in India where neonatal RV exposure was the most important determinant of post-vaccination anti-RV IgA (Parker et al., 2020). This confounding suggests that while anti-RV IgA seroconversion is a widely used correlate of RVV immunogenicity, it has limitations when used as a correlate of RVV protection against severe RVGE (Patel et al., 2013). Studies using disease endpoints (prevention of severe RVGE) rather than immunologic correlates to understand determinants of RVV protection are lacking and greatly needed.

Alongside the importance of naturally circulating *Reoviridae* in boosting anti-RV IgA, our work suggests that RVV immunogenicity in LMIC is significantly constrained by viral-viral interference of select *Picornaviridae* strains. *Picornaviridae* is a viral family consisting of 17 genera including *Enterovirus*, *Parechovirus*, *Salivirus*, and *Cosavirus* (Fauquet and Fargette, 2005). They are non-enveloped, positive-stranded RNA viruses that exhibit considerable genetic variability driven by both mutation and recombination (Zell, 2018). Several studies have shown that co-administration of OPV, an *Enterovirus C*, may lower RVV immunogenicity (Holtz et al., 2014; Parker et al., 2018b; Patel et al., 2012; Tan et al., 2020; Taniuchi et al., 2016). Inconsistent with those previous studies, the presence of poliovirus (*Enterovirus C*) was not significantly associated with RVV seroconversion in this study. Importantly, other *Picornaviridae* strains appeared to have more significant negative correlations with seroconversion. Specifically, the presence of *Enterovirus B* strains and several novel strains of *Cosavirus A* correlated negatively with seroconversion. The findings are consistent with reports that recent or concurrent infection with non-polio enterovirus is correlated with lower immunogenicity for both OPV and RVV (Cusick et al., 2014; Kernbauer et al., 2014; Parker et al., 2014; Praharaj et al., 2019; Taniuchi et al., 2016).

Unique from *Enterovirus B* and *C*, *Cosavirus* is a relatively underexplored genus of *Picornaviridae*. Cosaviruses have been identified in children with diarrheal illness in Africa, South America, and Asia but have not been identified as causative agents in acute gastroenteritis. Their true pathogenicity and disease burden remains poorly understood (Ayouni et al., 2016; Stöcker et al., 2012; Yu et al., 2017). In this study, multiple novel cosavirus contigs were detected, and novel *Cosavirus A* correlated significantly with lack of seroconversion at dose 1, suggesting a potential interaction between this poorly characterized family of viruses and RVV immunity.

The underappreciated abundance and richness of *Picornaviridae* strains in these asymptomatic Ghanaian infants suggest that orally administered RVVs is arriving in a complex intestinal milieu with almost certain viral co-infection. The mechanisms underlying a possible interference between *Picornaviridae* strains and RVVs are as yet unknown but may include viral competition or alteration of host receptors and cell entry, acute innate immune modulation, or innate immune training (Divangahi et al., 2021; Makimaa et al. 2020; Pinky and Dobrovolny, 2016; Raman et al.,2016; Ramani et al., 2018). Enteroviruses and other positive-strand RNA viruses can hijack host cell machinery, potentially outcompeting attenuated RVV strains (Drummond et al., 2017; Hsiung, 1961; Li et al., 2020). Alternatively, recent viral infection may modulate innate immunity to RVV. *In vitro* studies showed marked reduction of RV replication, coupled with altered IFN and cytokine expression, when RV was mixed with astroviruses or enteroviruses in cell culture (Wang et al., 2012). Finally, early viral exposures may train innate immunity to RVVs through epigenetic remodeling (Divangahi et al., 2021). This phenomenon has been shown for the live attenuated measles vaccine that can provide heterologous protection against unrelated pathogens in early life (Higgins et al., 2016; Netea et al., 2020).

Therefore, our data, which is derived from an unbiased, metagenomic approach, suggest that even previously unknown viral strains, such as novel cosaviruses, may have important roles in limiting RVV immunogenicity. However, our virome analysis had several key limitations. Analysis of the prokaryotic and eukaryotic virome is fundamentally limited by the significant number of viral reads that we were unable to assign, belonging to the "dark matter" of the virome (Li et al., 2021b). Additional biases may have been introduced by the use of VLPs and amplification procedures, but a major strength of our analysis was assessment of both RNA and DNA viromes (Gregory et al., 2020). A possible study limitation is that RVV administration is altering microbiome composition, potentially confounding microbiota associations with RVV immunogenicity. Previous studies have demonstrated negligible effects of RVVs on gut bacteriome composition (Ang et al., 2014; García-López et al., 2012), consistent with our bacteriome analysis from matched pre- and post-vaccination samples (Figures S1A and S1B). We were limited by the low number of pre-vaccination virome samples for dose 1 to evaluate whether the virome composition is similarly robust to any effects of RVVs.

There are public health implications of our work. We find that prokaryotic and eukaryotic viral interference may limit RVV performance in LMICs where the virome is likely richer and more diverse than the virome of infants from high-income countries. Improving RVV performance in the face of such interference may be complex, but two solutions may be possible. The first is moving RVV administration to earlier ages in LMICs, where there may be lower prokaryotic and eukaryotic viral carriage and potential for interference. Administration of RVVs in neonates has been shown in LMIC clinical trials to offer significantly higher protection than adminstration at later ages, suggesting this early administration may be an effective strategy to overcome viral interference (Bines et al., 2018). Alternatively, parenteral and non-oral RVVs may avoid enteric viral interference. The safety and efficacy of trivalent P2-VP8 subunit vaccine is currently being tested and may offer hope for improved protection against serious RVGE in LMICs (NCT04010448, clinicaltrials.gov) (Groome et al., 2020).

In conclusion, young Ghanaian infants have rich and complex prokaryotic and eukaryotic viromes, and bacteriophage diversity alongside known and novel *Picornaviridae* strains may be important adverse determinants of oral RVV immunogenicity. Our findings suggest that infants in LMICs may have earlier and more diverse viral infections than those in high-income countries and that RVV performance is likely limited by these enteric viral infections. Our work indicates that vaccination strategies such as neonatal vaccine administration or parenteral RVV administration are urgently needed to improve RVV protection in LMICs, where the morbidity and mortality of RV disease remains unacceptably high among young children.

STAR★METHODS

Detailed methods are provided in the online version of this paper and include the following:

- KEY RESOURCES TABLE
- RESOURCE AVAILABILITY
  - Lead contact
  - Materials availability
  - Data and code availability
- EXPERIMENTAL MODEL AND SUBJECT DETAILS
  - Study subjects and fecal samples
- METHOD DETAILS
  - Serum assays
  - Fecal sample collection
  - 16S rRNA gene amplicon sequencing
  - Bacteriome analysis
  - Viral-like particle preparation
  - Viral sequencing analysis
  - Metavirome assembly
  - Phylogenetic analysis
  - Phageome analysis
- QUANTIFICATION AND STATISTICAL ANALYSIS
  - Subject per dose averages

SUPPLEMENTAL INFORMATION

Supplemental information can be found online at https://doi.org/10.1016/j.chom.2021.12.002.

ACKNOWLEDGMENTS

We acknowledge and thank all the infants and their families who participated in this study and the staff members of the RVV trial team in Ghana for their work in conducting this study; the staff at the Noguchi Memorial Institute for Medical Research in Accra, Ghana; Monica McNeal and her staff at the Laboratory of Specialized Clinical Studies, Cincinnati Children's Hospital, who performed all the immunoglobulin testing for the original dosing studies; Duncan Steele for his contribution to the original dosing study in Ghana; and Lauren Gazley for her contribution to the original dosing study in Ghana. We are grateful to members of the Handley and Baldridge laboratories for helpful discussions. This was work was supported by a grant from the Bill and Melinda Gates Foundation (OPP1157923). M.T.B. was supported by R01 OD024917, R01 AI139314; Children's Discovery Institute of Washington University and St. Louis Children's Hospital Interdisciplinary Research Initiative grant (MI-II-2019-790); and the Mathers Foundation. A.H.K. was supported by T32 AI007163.

AUTHOR CONTRIBUTIONS

Conceptualization, V.C.H.; methodology, S.A.H., V.C.H., and G.A.; investigation, L.D., G.A., and F.D.; software, A.H.K., S.A.H., L.W., and R.R.; formal analysis, A.H.K. and S.A.H.; resources, G.A., F.D., S.A.H., and M.T.B.; writing – original draft, A.H.K. and V.C.H.; writing – review and editing, A.H.K., V.C.H., M.T.B., S.A.H., F.D., and G.A.; funding acquisition, V.C.H.; visualization, A.H.K.; supervision, M.T.B., S.A.H., and V.C.H.

DECLARATION OF INTERESTS

The authors declare no competing interests.

Received: June 4, 2021
Revised: October 22, 2021
Accepted: November 29, 2021
Published: December 20, 2021

REFERENCES

Altan, E., Aiemjoy, K., Phan, T.G., Deng, X., Aragie, S., Tadesse, Z., Callahan, K.E., Keenan, J., and Delwart, E. (2018). Enteric virome of Ethiopian children participating in a clean water intervention trial. PLoS One *13*, e0202054.

Ang, L., Arboleya, S., Lihua, G., Chuihui, Y., Nan, Q., Suarez, M., Solís, G., De Los Reyes-Gavilán, C.G., and Gueimonde, M. (2014). The establishment of the infant intestinal microbiome is not affected by rotavirus vaccination. Sci. Rep. *4*, 7417.

Armah, G., Lewis, K.D.C., Cortese, M.M., Parashar, U.D., Ansah, A., Gazley, L., Victor, J.C., McNeal, M.M., Binka, F., and Steele, A.D. (2016). A randomized, controlled trial of the impact of alternative dosing schedules on the immune response to human rotavirus vaccine in rural Ghanaian infants. J. Infect. Dis. *213*, 1678–1685.

Atherly, D., Dreibelbis, R., Parashar, U.D., Levin, C., Wecker, J., and Rheingans, R.D. (2009). Rotavirus vaccination: cost-effectiveness and impact on child mortality in developing countries. J. Infect. Dis. *200* (suppl 1), S28–S38.

Ayouni, S., Estienney, M., Hammami, S., Neji Guediche, M., Pothier, P., Aouni, M., Belliot, G., and de Rougemont, A. (2016). Cosavirus, Salivirus and Bufavirus in Diarrheal Tunisian Infants. PLoS One *11*, e0162255.

Baldridge, M.T., Nice, T.J., McCune, B.T., Yokoyama, C.C., Kambal, A., Wheadon, M., Diamond, M.S., Ivanova, Y., Artyomov, M., and Virgin, H.W. (2015). Commensal microbes and interferon-λ determine persistence of enteric murine norovirus infection. Science *347*, 266–269.

Belkaid, Y., and Harrison, O.J. (2017). Homeostatic immunity and the microbiota. Immunity *46*, 562–576.

Bernstein, D.I., Smith, V.E., Sherwood, J.R., Schiff, G.M., Sander, D.S., DeFeudis, D., Spriggs, D.R., and Ward, R.L. (1998). Safety and immunogenicity of live, attenuated human rotavirus vaccine 89-12. Vaccine *16*, 381–387.

Bines, J.E., At Thobari, J., Satria, C.D., Handley, A., Watts, E., Cowley, D., Nirwati, H., Ackland, J., Standish, J., Justice, F., et al. (2018). Human neonatal rotavirus vaccine (RV3-BB) to target rotavirus from birth. N. Engl. J. Med. *378*, 719–730.

Bray, N.L., Pimentel, H., Melsted, P., and Pachter, L. (2016). Near-optimal probabilistic RNA-seq quantification. Nat. Biotechnol. *34*, 525–527.

Callahan, B.J., McMurdie, P.J., Rosen, M.J., Han, A.W., Johnson, A.J.A., and Holmes, S.P. (2016). DADA2: high-resolution sample inference from Illumina amplicon data. Nat. Methods *13*, 581–583.

Caporaso, J.G., Lauber, C.L., Walters, W.A., Berg-Lyons, D., Lozupone, C.A., Turnbaugh, P.J., Fierer, N., and Knight, R. (2011). Global patterns of 16S rRNA diversity at a depth of millions of sequences per sample. Proc. Natl. Acad. Sci. U. S. A. *108* (suppl 1), 4516–4522.

Church, J.A., Parker, E.P., Kirkpatrick, B.D., Grassly, N.C., and Prendergast, A.J. (2019a). Interventions to improve oral vaccine performance: a systematic review and meta-analysis. Lancet Infect. Dis. *19*, 203–214.

Church, J.A., Rukobo, S., Govha, M., Lee, B., Carmolli, M.P., Chasekwa, B., Ntozini, R., Mutasa, K., McNeal, M.M., Majo, F.D., et al. (2019b). The impact of improved water, sanitation, and hygiene on oral rotavirus vaccine immunogenicity in Zimbabwean infants: substudy of a cluster-randomized trial. Clin. Infect. Dis. *69*, 2074–2081.

Cole, J.R., Wang, Q., Fish, J.A., Chai, B., McGarrell, D.M., Sun, Y., Brown, C.T., Porras-Alfaro, A., Kuske, C.R., and Tiedje, J.M. (2014). Ribosomal Database Project: data and tools for high throughput rRNA analysis. Nucleic Acids Res *42*, D633–D642.

Cowley, D., Boniface, K., Bogdanovic-Sakran, N., Kirkwood, C.D., and Bines, J.E. (2017). Rotavirus shedding following administration of RV3-BB human neonatal rotavirus vaccine. Hum. Vaccin. Immunother. *13*, 1908–1915.

Cusick, M.F., Libbey, J.E., and Fujinami, R.S. (2014). Picornavirus infection leading to immunosuppression. Future Virol *9*, 475–482.

Dallari, S., Heaney, T., Rosas-Villegas, A., Neil, J.A., Wong, S.-Y., Brown, J.J., Urbanek, K., Herrmann, C., Depledge, D.P., Dermody, T.S., and Cadwell, K. (2021). Enteric viruses evoke broad host immune responses resembling those elicited by the bacterial microbiome. Cell Host Microbe *29*, 1014–1029.e8.

CellPress
OPEN ACCESS

Divangahi, M., Aaby, P., Khader, S.A., Barreiro, L.B., Bekkering, S., Chavakis, T., Crevel, R. van, Curtis, N., DiNardo, A.R., Dominguez-Andres, J., et al. (2021). Trained immunity, tolerance, priming and differentiation: distinct immunological processes. Nat. Immunol. *22*, 2–6.

Drummond, C.G., Bolock, A.M., Ma, C., Luke, C.J., Good, M., and Coyne, C.B. (2017). Enteroviruses infect human enteroids and induce antiviral signaling in a cell lineage-specific manner. Proc. Natl. Acad. Sci. U. S. A. *114*, 1672–1677.

Edgar, R.C. (2004). MUSCLE: multiple sequence alignment with high accuracy and high throughput. Nucleic Acids Res *32*, 1792–1797.

Elbers, B., and Oldoni, D.. https://cran.r-project.org/web/packages/tidylog/index.html.

Erickson, A.K., Jesudhasan, P.R., Mayer, M.J., Narbad, A., Winter, S.E., and Pfeiffer, J.K. (2018). Bacteria facilitate enteric virus co-infection of mammalian cells and promote genetic recombination. Cell Host Microbe *23*, 77–88.e5.

Fauquet, C.M., and Fargette, D. (2005). International Committee on Taxonomy of Viruses and the 3,142 unassigned species. Virol. J. *2*, 64.

Federici, S., Nobs, S.P., and Elinav, E. (2021). Phages and their potential to modulate the microbiome and immunity. Cell. Mol. Immunol. *18*, 889–904.

Finkbeiner, S.R., Holtz, L.R., Jiang, Y., Rajendran, P., Franz, C.J., Zhao, G., Kang, G., and Wang, D. (2009). Human stool contains a previously unrecognized diversity of novel astroviruses. Virol. J. *6*, 161.

Fix, J., Chandrashekhar, K., Perez, J., Bucardo, F., Hudgens, M.G., Yuan, L., Twitchell, E., Azcarate-Peril, M.A., Vilchez, S., and Becker-Dreps, S. (2020). Association between gut microbiome composition and rotavirus vaccine response among Nicaraguan infants. Am. J. Trop. Med. Hyg. *102*, 213–219.

García-López, R., Pérez-Brocal, V., Diez-Domingo, J., and Moya, A. (2012). Gut microbiota in children vaccinated with rotavirus vaccine. Pediatr. Infect. Dis. J. *31*, 1300–1302.

Garnier, S., Ross, N., Rudis, B., Sciaini, M., Camargo, A.P., and Scherer, C. (2021). viridis: Colorblind-Friendly Color Maps for R (R). https://cran.r-project.org/web/packages/viridis/index.html.

Gascuel, O. (1997). BIONJ: an improved version of the NJ algorithm based on a simple model of sequence data. Mol. Biol. Evol. *14*, 685–695.

GBD 2016 Causes of Death Collaborators (2017). Global, regional, and national age-sex specific mortality for 264 causes of death, 1980–2016: a systematic analysis for the Global Burden of Disease Study 2016. Lancet *390*, 1151–1210.

GBD 2016 Diarrhoeal Disease Collaborators (2018). Estimates of the global, regional, and national morbidity, mortality, and aetiologies of diarrhoea in 195 countries: a systematic analysis for the Global Burden of Disease Study 2016. Lancet Infect. Dis. *18*, 1211–1228.

Ghana Statistical Service. (2015). Ghana health Service, and ICF International (2015). Ghana Demogr. Health Surv.

Gogokhia, L., Buhrke, K., Bell, R., Hoffman, B., Brown, D.G., Hanke-Gogokhia, C., Ajami, N.J., Wong, M.C., Ghazaryan, A., Valentine, J.F., et al. (2019). Expansion of bacteriophages is linked to aggravated intestinal inflammation and colitis. Cell Host Microbe *25*, 285–299.e8.

Grassly, N.C., Praharaj, I., Babji, S., Kaliappan, S.P., Giri, S., Venugopal, S., Parker, E.P.K., Abraham, A., Muliyil, J., Doss, S., et al. (2016). The effect of azithromycin on the immunogenicity of oral poliovirus vaccine: a double-blind randomised placebo-controlled trial in seronegative Indian infants. Lancet Infect. Dis. *16*, 905–914.

Gregory, A.C., Zablocki, O., Zayed, A.A., Howell, A., Bolduc, B., and Sullivan, M.B. (2020). The gut virome database reveals age-dependent patterns of virome diversity in the human gut. Cell Host Microbe *28*, 724–740.e8.

Groome, M.J., Fairlie, L., Morrison, J., Fix, A., Koen, A., Masenya, M., Jose, L., Madhi, S.A., Page, N., McNeal, M., et al. (2020). Safety and immunogenicity of a parenteral trivalent P2-VP8 subunit rotavirus vaccine: a multisite, randomised, double-blind, placebo-controlled trial. Lancet Infect. Dis. *20*, 851–863.

Harris, V., Ali, A., Fuentes, S., Korpela, K., Kazi, M., Tate, J., Parashar, U., Wiersinga, W.J., Giaquinto, C., de Weerth, C., et al. (2018a). Rotavirus vaccine response correlates with the infant gut microbiota composition in Pakistan. Gut Microbes *9*, 93–101.

Harris, V.C., Armah, G., Fuentes, S., Korpela, K.E., Parashar, U., Victor, J.C., Tate, J., de Weerth, C., Giaquinto, C., Wiersinga, W.J., and de Vos, V.M. (2017). Significant correlation Between the infant gut microbiome and rotavirus vaccine response in rural Ghana. J. Infect. Dis. *215*, 34–41.

Harris, V.C., Haak, B.W., Handley, S.A., Jiang, B., Velasquez, D.E., Hykes, B.L., Droit, L., Berbers, G.A.M., Kemper, E.M., van Leeuwen, E.M.M., et al. (2018b). Effect of antibiotic-mediated microbiome modulation on rotavirus vaccine immunogenicity: a human, randomized-control proof-of-concept trial. Cell Host Microbe *24*, 197–207.e4.

Higgins, J.P.T., Soares-Weiser, K., López-López, J.A., Kakourou, A., Chaplin, K., Christensen, H., Martin, N.K., Sterne, J.A.C., and Reingold, A.L. (2016). Association of BCG, DTP, and measles containing vaccines with childhood mortality: systematic review. BMJ *355*, i5170.

Holtz, L.R., Cao, S., Zhao, G., Bauer, I.K., Denno, D.M., Klein, E.J., Antonio, M., Stine, O.C., Snelling, T.L., Kirkwood, C.D., and Wang, D. (2014). Geographic variation in the eukaryotic virome of human diarrhea. Virology *468–470*, 556–564.

Hsiung, G.D. (1961). Multiplication of poliovirus in tissue cultures previously infected with other enteroviruses. Arch. Gesamte Virusforsch *11*, 343–354.

Jones, D.T., Taylor, W.R., and Thornton, J.M. (1992). The rapid generation of mutation data matrices from protein sequences. Comput. Appl. Biosci. *8*, 275–282.

Jonesteller, C.L., Burnett, E., Yen, C., Tate, J.E., and Parashar, U.D. (2017). Effectiveness of rotavirus vaccination: a systematic review of the first decade of global postlicensure data, 2006–2016. Clin. Infect. Dis. *65*, 840–850.

Kassambara, A.. https://cran.r-project.org/web/packages/ggpubr/index.html.

Kassambara, A.. https://cran.r-project.org/web/packages/rstatix/index.html.

Kernbauer, E., Ding, Y., and Cadwell, K. (2014). An enteric virus can replace the beneficial function of commensal bacteria. Nature *516*, 94–98.

Kim, A.H.J., Hogarty, M.P., Harris, V.C., and Baldridge, M.T. (2021). The complex interactions between rotavirus and the gut microbiota. Front. Cell. Infect. Microbiol. *10*, 586751.

Kolmogorov, M., Bickhart, D.M., Behsaz, B., Gurevich, A., Rayko, M., Shin, S.B., Kuhn, K., Yuan, J., Polevikov, E., Smith, T.P.L., and Pevzner, P.A. (2020). metaFlye: scalable long-read metagenome assembly using repeat graphs. Nat. Methods *17*, 1103–1110.

Kumar, S., Stecher, G., Li, M., Knyaz, C., and Tamura, K. (2018). MEGA X: molecular evolutionary genetics analysis across computing platforms. Mol. Biol. Evol. *35*, 1547–1549.

Kuss, S.K., Best, G.T., Etheredge, C.A., Pruijssers, A.J., Frierson, J.M., Hooper, L.V., Dermody, T.S., and Pfeiffer, J.K. (2011). Intestinal microbiota promote enteric virus replication and systemic pathogenesis. Science *334*, 249–252.

Li, D., Liu, C.-M., Luo, R., Sadakane, K., and Lam, T.-W. (2015). MEGAHIT: an ultra-fast single-node solution for large and complex metagenomics assembly via succinct de Bruijn graph. Bioinformatics *31*, 1674–1676.

Li, X., Mukandavire, C., Cucunubá, Z.M., Echeverria Londono, S., Abbas, K., Clapham, H.E., Jit, M., Johnson, H.L., Papadopoulos, T., Vynnycky, E., et al. (2021a). Estimating the health impact of vaccination against ten pathogens in 98 low-income and middle-income countries from 2000 to 2030: a modelling study. Lancet *397*, 398–408.

Li, X., Wang, M., Cheng, A., Wen, X., Ou, X., Mao, S., Gao, Q., Sun, D., Jia, R., Yang, Q., et al. (2020). Enterovirus replication organelles and inhibitors of their formation. Front. Microbiol. *11*, 1817.

Li, Y., Handley, S.A., and Baldridge, M.T. (2021b). The dark side of the gut: virome-host interactions in intestinal homeostasis and disease. J. Exp. Med. *218*, e20201044.

Liang, G., and Bushman, F.D. (2021). The human virome: assembly, composition and host interactions. Nat. Rev. Microbiol. *19*, 514–527.

Liang, G., Zhao, C., Zhang, H., Mattei, L., Sherrill-Mix, S., Bittinger, K., Kessler, L.R., Wu, G.D., Baldassano, R.N., DeRusso, P., et al. (2020). The stepwise assembly of the neonatal virome is modulated by breastfeeding. Nature *581*, 470–474.

Lim, E.S., Wang, D., and Holtz, L.R. (2016). The bacterial microbiome and virome milestones of infant development. Trends Microbiol *24*, 801–810.

Lim, E.S., Zhou, Y., Zhao, G., Bauer, I.K., Droit, L., Ndao, I.M., Warner, B.B., Tarr, P.I., Wang, D., and Holtz, L.R. (2015). Early life dynamics of the human gut virome and bacterial microbiome in infants. Nat. Med. *21*, 1228–1234.

Love, M.I., Huber, W., and Anders, S. (2014). Moderated estimation of fold change and dispersion for RNA-seq data with DESeq2. Genome Biol *15*, 550.

Makimaa, H., Ingle, H., and Baldridge, M.T. (2020). Enteric viral co-infections: pathogenesis and perspective. Viruses *12*, 904.

Maqsood, R., Rodgers, R., Rodriguez, C., Handley, S.A., Ndao, I.M., Tarr, P.I., Warner, B.B., Lim, E.S., and Holtz, L.R. (2019). Discordant transmission of bacteria and viruses from mothers to babies at birth. Microbiome *7*, 156.

McMurdie, P.J., and Holmes, S. (2013). phyloseq: an R package for reproducible interactive analysis and graphics of microbiome census data. PLoS One *8*, e61217.

Mogotsi, M.T., Mwangi, P.N., Bester, P.A., Mphahlele, M.J., Seheri, M.L., O'Neill, H.G., and Nyaga, M.M. (2020). Metagenomic analysis of the enteric RNA virome of infants from the Oukasie Clinic, North West Province, South Africa, reveals diverse eukaryotic viruses. Viruses *12*, 1260.

Neil, J.A., and Cadwell, K. (2018). The intestinal virome and immunity. J. Immunol. *201*, 1615–1624.

Netea, M.G., Domínguez-Andrés, J., Barreiro, L.B., Chavakis, T., Divangahi, M., Fuchs, E., Joosten, L.A.B., van der Meer, J.W.M., Mhlanga, M.M., Mulder, W.J.M., et al. (2020). Defining trained immunity and its role in health and disease. Nat. Rev. Immunol. *20*, 375–388.

Neuwirth, E.. https://cran.r-project.org/web/packages/RColorBrewer/index.html.

Norman, J.M., Handley, S.A., Baldridge, M.T., Droit, L., Liu, C.Y., Keller, B.C., Kambal, A., Monaco, C.L., Zhao, G., Fleshner, P., et al. (2015). Disease-specific alterations in the enteric virome in inflammatory bowel disease. Cell *160*, 447–460.

Parker, E.P., Ramani, S., Lopman, B.A., Church, J.A., Iturriza-Gómara, M., Prendergast, A.J., and Grassly, N.C. (2018a). Causes of impaired oral vaccine efficacy in developing countries. Future Microbiol *13*, 97–118.

Oksanen, J., Blanchet, G., Friendly, M., Kindt, R., Legendre, P., McGlinn, D., Minchin, P.R., O'Hara, R.B., Simpson, G.L., Solymos, P., et al.. https://cran.r-project.org/web/packages/vegan/index.html.

Parker, E.P.K., Bronowski, C., Sindhu, K.N.C., Babji, S., Benny, B., Carmona-Vicente, N., Chasweka, N., Chinyama, E., Cunliffe, N.A., Dube, Q., et al. (2020). Impact of maternal antibodies and microbiota development on the immunogenicity of oral rotavirus vaccine in African, Indian, and European infants: A prospective cohort study. MedRxiv.

Parker, E.P.K., Kampmann, B., Kang, G., and Grassly, N.C. (2014). Influence of enteric infections on response to oral poliovirus vaccine: a systematic review and meta-analysis. J. Infect. Dis. *210*, 853–864.

Parker, E.P.K., Praharaj, I., Zekavati, A., Lazarus, R.P., Giri, S., Operario, D.J., Liu, J., Houpt, E., Iturriza-Gómara, M., Kampmann, B., et al. (2018b). Influence of the intestinal microbiota on the immunogenicity of oral rotavirus vaccine given to infants in south India. Vaccine *36*, 264–272.

Patel, M., Glass, R.I., Jiang, B., Santosham, M., Lopman, B., and Parashar, U. (2013). A systematic review of anti-rotavirus serum IgA antibody titer as a potential correlate of rotavirus vaccine efficacy. J. Infect. Dis. *208*, 284–294.

Patel, M., Steele, A.D., and Parashar, U.D. (2012). Influence of oral polio vaccines on performance of the monovalent and pentavalent rotavirus vaccines. Vaccine *30* (suppl 1), A30–A35.

Pfeiffer, J.K., and Virgin, H.W. (2016). Viral immunity. Transkingdom control of viral infection and immunity in the mammalian intestine. Science *351*, aad5872.

Pinky, L., and Dobrovolny, H.M. (2016). Coinfections of the respiratory tract: viral competition for resources. PLoS One *11*, e0155589.

Praharaj, I., Parker, E.P.K., Giri, S., Allen, D.J., Silas, S., Revathi, R., Kaliappan, S.P., John, J., Prasad, J.H., Kampmann, B., et al. (2019). Influence of nonpolio enteroviruses and the bacterial gut microbiota on oral poliovirus vaccine response: a study from South India. J. Infect. Dis. *219*, 1178–1186.

Raman, R., Tharakaraman, K., Sasisekharan, V., and Sasisekharan, R. (2016). Glycan-protein interactions in viral pathogenesis. Curr. Opin. Struct. Biol. *40*, 153–162.

Ramani, S., Stewart, C.J., Laucirica, D.R., Ajami, N.J., Robertson, B., Autran, C.A., Shinge, D., Rani, S., Anandan, S., Hu, L., et al. (2018). Human milk oligosaccharides, milk microbiome and infant gut microbiome modulate neonatal rotavirus infection. Nat. Commun. *9*, 5010.

Rampelli, S., Turroni, S., Schnorr, S.L., Soverini, M., Quercia, S., Barone, M., Castagnetti, A., Biagi, E., Gallinella, G., Brigidi, P., and Candela, M. (2017). Characterization of the human DNA gut virome across populations with different subsistence strategies and geographical origin. Environ. Microbiol. *19*, 4728–4735.

Reyes, A., Blanton, L.V., Cao, S., Zhao, G., Manary, M., Trehan, I., Smith, M.I., Wang, D., Virgin, H.W., Rohwer, F., and Gordon, J.I. (2015). Gut DNA viromes of Malawian twins discordant for severe acute malnutrition. Proc. Natl. Acad. Sci. U. S. A. *112*, 11941–11946.

Reyes, A., Wu, M., McNulty, N.P., Rohwer, F.L., and Gordon, J.I. (2013). Gnotobiotic mouse model of phage-bacterial host dynamics in the human gut. Proc. Natl. Acad. Sci. U. S. A. *110*, 20236–20241.

Robinson, C.M., Jesudhasan, P.R., and Pfeiffer, J.K. (2014). Bacterial lipopolysaccharide binding enhances virion stability and promotes environmental fitness of an enteric virus. Cell Host Microbe *15*, 36–46.

PATH, Centers for Disease Control and Prevention (CDC), and World Health Organization (WHO). (2006). Accelerating the introduction of rotavirus vaccines into GAVI-eligible countries.

ROTA Council. (2020). Global introduction status.

Sindhu, K.N.C., Cunliffe, N., Peak, M., Turner, M., Darby, A., Grassly, N., Gordon, M., Dube, Q., Babji, S., Praharaj, I., et al. (2017). Impact of maternal antibodies and infant gut microbiota on the immunogenicity of rotavirus vaccines in African, Indian and European infants: protocol for a prospective cohort study. BMJ Open *7*, e016577.

Smith, S.D. (2019). phylosmith: an R-package for reproducible and efficient microbiome analysis with phyloseq-objects. J. Open Source SoftW. *4*, 38.

Stefan, K.L., Kim, M.V., Iwasaki, A., and Kasper, D.L. (2020). Commensal microbiota modulation of natural resistance to virus infection. Cell *183*, 1312–1324.e10.

Steinegger, M., and Söding, J. (2017). MMseqs2 enables sensitive protein sequence searching for the analysis of massive data sets. Nat. Biotechnol. *35*, 1026–1028.

Stöcker, A., Souza, B.F., Ribeiro, T.C., Netto, E.M., Araujo, L.O., Corrêa, J.I., Almeida, P.S., de Mattos, A.P., Ribeiro, Hda C., Pedral-Sampaio, D.B., et al. (2012). Cosavirus infection in persons with and without gastroenteritis. Brazil. Emerg. Infect. Dis. *18*, 656–659.

Tan, S.K., Granados, A.C., Bouquet, J., Hoy-Schulz, Y.E., Green, L., Federman, S., Stryke, D., Haggerty, T.D., Ley, C., Yeh, M.-T., et al. (2020). Metagenomic sequencing of stool samples in Bangladeshi infants: virome association with poliovirus shedding after oral poliovirus vaccination. Sci. Rep. *10*, 15392.

Taniuchi, M., Platts-Mills, J.A., Begum, S., Uddin, M.J., Sobuz, S.U., Liu, J., Kirkpatrick, B.D., Colgate, E.R., Carmolli, M.P., Dickson, D.M., et al. (2016). Impact of enterovirus and other enteric pathogens on oral polio and rotavirus vaccine performance in Bangladeshi infants. Vaccine *34*, 3068–3075.

Tate, J.E., Burton, A.H., Boschi-Pinto, C., and Parashar, U.D.; World Health Organization–Coordinated Global Rotavirus Surveillance Network (2016). Global, regional, and national estimates of rotavirus mortality in children <5 years of age, 2000–2013. Clin. Infect. Dis. *62* (suppl 2), S96–S105.

Troeger, C., Khalil, I.A., Rao, P.C., Cao, S., Blacker, B.F., Ahmed, T., Armah, G., Bines, J.E., Brewer, T.G., Colombara, D.V., et al. (2018). Rotavirus vaccination and the global burden of rotavirus diarrhea among children younger than 5 years. JAMA Pediatr *172*, 958–965.

Wang, D. (2020). 5 challenges in understanding the role of the virome in health and disease. PLoS Pathog *16*, e1008318.

Wang, H., Moon, S., Wang, Y., and Jiang, B. (2012). Multiple virus infection alters rotavirus replication and expression of cytokines and toll-like receptors in intestinal epithelial cells. Virus Res *167*, 48–55.

Ward, R.L., Bernstein, D.I., Shukla, R., McNeal, M.M., Sherwood, J.R., Young, E.C., and Schiff, G.M. (1990). Protection of adults rechallenged with a human rotavirus. J. Infect. Dis. *161*, 440–445.

Wickham, H.. https://cran.r-project.org/web/packages/reshape/index.html.

Wickham, H.. https://cran.r-project.org/web/packages/tidyverse/index.html.

Wickham, H., François, R., Henry, L., and Müller, K.. https://cran.r-project.org/web/packages/dplyr/index.html.

Xie, Y., Sarma, A., Vogt, A., Andrew, A., Zvoleff, A., Simon, A., Atkins, A., Wolen, A., Manton, A., Yasumoto, A., et al.. https://cran.r-project.org/web/packages/knitr/index.html.

Yu, J.-M., Ao, Y.-Y., Li, L.-L., and Duan, Z.-J. (2017). Identification of a novel cosavirus species in faeces of children and its relationship with acute gastroenteritis in China. Clin. Microbiol. Infect. *23*, 550–554.

Zell, R. (2018). Picornaviridae-the ever-growing virus family. Arch. Virol. *163*, 299–317.

Csárdi, G., Hester, J., Wickham, H., Chang, W., Morgan, M., Tenenbaum, D. (2021). remotes: R Package Installation from Remote Repositories, Including 'GitHub'. https://cran.r-project.org/web/packages/remotes/index.html.

Dowle, M., Srinivasan, A., Gorecki, J., Chirico, M., Stetsenko, P., Short, T., Lianoglou, S., Antonyan, E., Bonsch, M., Parsonage, H., et al. (2021). data.table: Extension of 'data.frame'. https://cran.r-project.org/web/packages/data.table/index.html.

Wickham, H., Chang, W., Henry, L., Pedersen, T.L., Takahashi, K., Wilke, C., Woo, K., Yutani, H., Dunnington, D. (2021). ggplot2: Create Elegant Data Visualisations Using the Grammar of Graphics. (R Project). https://cran.r-project.org/web/packages/ggplot2/index.html.

Hester, J. (2020). glue: Interpreted String Literals. (Microsoft). https://mran.microsoft.com/snapshot/2020-05-03/web/packages/glue/index.html.

Slowikowski, K., Schep, A., Hughes, S., Dang, T.K., Lukauskas, S., Irisson, J.O., Kamvar, Z.N., Ryan, T., Christophe, D., Hiroaki, Y., et al. (2021). ggrepel: Automatically Position Non-Overlapping Text Labels with 'ggplot2'. https://cran.r-project.org/web/packages/ggrepel/index.html.

STAR★METHODS

KEY RESOURCES TABLE

REAGENT or RESOURCE	SOURCE	IDENTIFIER
Antibodies		
biotinylated goat anti human IgA	Jackson Laboratories	Cat# 109-005-011; RRID: AB_2337535
hyperimmune rabbit serum made from rabbits immunized with several different purified rotavirus strains	Laboratory of Specialized Clinical Studies, Cincinnati Children's Hospital	N/A
Bacterial and virus strains		
rotavirus lysate strain 89-12, a G1P8 strain that was used to develop Rotarix ® (GlasxoSmithKline),	Laboratory of Specialized Clinical Studies, Cincinnati Children's Hospital	N/A
Biological samples		
460 fecal samples	Rotarix trial, NCT01575197, clinicaltrials.gov	N/A
122 serum samples, anti-RV IgA analysis performed previously at Cincinnati Children's Hospital Medical Center Laboratory for Specialized Clinical Studies (Cincinnati, Ohio, USA) by enzyme-linked immunoassay (ELISA) as previously described to detect and quantify serum anti-rotavirus IgA or IgG antibody concentrations (U/mL).	Rotarix trial, NCT01575 197, clinicaltrials.gov, Ward et al., 1990, Bernstein et al., 1998	N/A
Chemicals, peptides, and recombinant proteins		
Total Nucleic Acid Isolation Kit	Roche Diagnostics	Cat# 3337928190
100mM dNTP Set	Fisher Scientific	Cat# 10297018
M MLV Reverse Transcriptase	Fisher Scientific	Cat# PRM1701
Sequenase V2.0 T7 DNA Pol (1000 UN)	Fisher Scientific	Cat# 70775Z
AccuPrime Taq DNA Polymerase System	Fisher Scientific	Cat# 12339016
Qubit dsDNA HS Assay Kit	Life Technologies	Cat# Q32851
NEBNext Ultra DNA Library Prep Kit for Illumina - 96 rxns	New England Biolabs	Cat# E7370L
NEBNext Multiplex Oligos for Illumina (Index Primers Set 1) - 24 rxns	New England Biolabs	Cat# E7335S
NEBNext Multiplex Oligos for Illumina (Index Primers Set 2) - 24 rxns	New England Biolabs	Cat# E7500S
NEBNext Multiplex Oligos for Illumina (Index Primers Set 3) - 24 rxns	New England Biolabs	Cat# E7710S
NEBNext Multiplex Oligos for Illumina (Index Primers Set 4) - 24 rxns	New England Biolabs	Cat# E7730S
Agencourt AMPure XP 60mL	Beckman Coulter	Cat# A63881
Agilent High Sensitivity DNA Kit	Agilent Technologies	Cat# 5067-4626
DNeasy 96 Blood & Tissue Kit (4)	Qiagen	Cat# 69581
Platinum Taq High Fidelity	Fisher Scientific	Cat# 11304029
DNTP Mix	Fisher Scientific	Cat# PRU1515
Agencourt AMPure XP 60mL	Beckman Coulter	Cat# A63881
Agilent High Sensitivity DNA Kit	Agilent Technologies	Cat# 5067-4626
peroxidase conjugated avidin:biotin	Vector Laboratories	Cat# A-2014-5
substrate O-phenylenediamine (OPD)	Sigma Aldrich	Ca# P9029

(*Continued on next page*)

CellPress
OPEN ACCESS

Continued

REAGENT or RESOURCE	SOURCE	IDENTIFIER
Deposited data		
16S rRNA sequencing data	This paper	European Nucleic Acid Archive, ENA: PRJEB39845
Unprocessed virome sequencing data	This paper	European Nucleic Acid Archive, ENA: PRJEB39845
Full analysis workflows for microbiome, virome, dada2 ASV resolution, statistical analysis and plotting	This paper	Zenodo: https://doi.org/10.5281/zenodo.5711741; Github: https://github.com/shandley/rotabiome
Oligonucleotides		
Read 1 Sequencing Primer, TATGGTAA TTGTGTGCCAGCMGCCGCGGTAA	This paper	N/A
Read 2 Sequencing Primer, AGTCAGTC AGCCGGACTACHVGGGTWTCTAAT	This paper	N/A
Index Sequence Primer, ATTAGAWACC CBDGTAGTCCGGCTGACTGACT	This paper	N/A
Primer For PCR, AATGATACGGCGACCA CCGAGATCTACACTATGGTAATTGTGTG CCAGCMGCCGCGGTAA	This paper	N/A
Software and algorithms		
GraphPad Prism 9	GraphPad San Diego, CA	Version 9.2.0; RRID: SCR_002798
Rstudio	RStudio, Inc	Version 1.0.143; RRID: SCR_00432
MegaHit assembler	Li et al., 2015	MegaHit, RRID: SCR_018551
DESeq2	Love et al., 2014	DESeq2, RRID: SCR_015687
MMseqs2	Steinegger and Söding., 2017	https://github.com/soedinglab/MMseqs2
Phyloseq	McMurdie and Holmes., 2013	phyloseq, RRID: SCR_013080
RcolorBrewer	(Neuwirth, 2014)	RColorBrewer, RRID: SCR_016697
Vegan	(Oksanen et al., 2021)	vegan, RRID: SCR_011950
Knitr	(Xie et al., 2021)	knitr, RRID: SCR_018533
Viridis	(Garnier et al., 2021)	viridis, RRID: SCR_016696
Rstatix	(Kassambara, 2021)	rstatix, RRID: SCR_021240
Remotes	(Csárdi et al., 2021)	https://cran.r-project.org/web/packages/remotes/index.html
Phylosmith	(Smith, 2019)	https://github.com/schuyler-smith/phylosmith
Reshape	(Wickham, 2018)	reshape, RRID: SCR_018983
Dada2	Callahan et al., 2016	https://github.com/benjjneb/dada2; RRID: SCR_008205
Tidyverse	(Wickham, 2021)	tidyverse, RRID: SCR_019186
Ggpubr	(Kassambara, 2020)	ggpubr, RRID: SCR_008205
Data.table	(Dowle et al., 2021)	https://github.com/Rdatatable/data.table
ggplot2	(Wickham et al., 2021)	ggplot2, RRID: SCR_014601
Dplyr	(Wickham et al., 2021)	dplyr, RRID: SCR_016708
Tidylog	(Elbers and Oldoni, 2020)	https://github.com/elbersb/tidylog/
Glue	(Hester, 2020)	https://glue.tidyverse.org/
Ggrepel	(Slowikowski et al., 2021)	ggrepel, RRID: SCR_017393

RESOURCE AVAILABILITY

Lead contact

Further information and requests for resources and reagents should be directed to and will be fulfilled by the lead contact, Vanessa C Harris (v.c.harris@amsterdamumc.nl).

Materials availability

This study did not generate new unique reagents.

Data and code availability

- All sequencing (16S rRNA and unprocessed virome sequence) data has been deposited to the European Nucleic Acid Archive (ENA) under project PRJEB39845 and are publicly available as of the date of the publication. Accession numbers are listed in the key resources table.
- All original code and a fully reproducible workflow for sequence processing as well as the analysis presented in this manuscript and has been deposited at Zenodo and are publicly available as of the date of the publication. DOI is listed in the key resources table.
- Any additional information required to reanalyze the data reported in this work paper is available from the Lead Contact upon request.

EXPERIMENTAL MODEL AND SUBJECT DETAILS

Study subjects and fecal samples

This study was nested in a previously-reported phase IV randomized clinical trial evaluating the immunogenicity of the Rotarix® vaccine after different dosing schedules (at age 6 and 10 weeks, 10 and 14 weeks, or 6, 10, and 14 weeks) (NCT01575197, clinicaltrials.gov) (Armah et al., 2016). The original trial was conducted in Navrongo, a rural district in northern Ghana where >70% of the population belong to the lowest wealth quintile in Ghana. The infant infant and under-5 mortality rates in northern Ghana at the time of the study, in 2012, were 5341 and 111 deaths per 1,000 live births, respectively (Ghana Statistical Service, 2015). All participating infants were healthy with a birth weight of > 2000 g and/or a gestational age > 38 weeks. Females and males were equally recruited and represented in the study (Table 1).

METHOD DETAILS

Serum assays

Sera from the original trial were analyzed at Cincinnati Children's Hospital Medical Center Laboratory for Specialized Clinical Studies (Cincinnati, Ohio, USA) by enzyme-linked immunoassay (ELISA) to detect anti-RV IgA antibody titer as described previously, with values expressed in international units per milliliter (Armah et al., 2016; Bernstein et al., 1998; Ward et al., 1990). Briefly, 96-well EIA plates (Costar, (Fisher Scientific, Pittsburgh, PA)) were coated (100 μl well) with a 1:1,000 dilution of hyperimmune rabbit serum made in house from rabbits immunized with several different purified rotavirus strains . After overnight incubation at 4°C, the plates were washed and rotavirus lysate strain 89-12, a G1P8 strain that was used to develop Rotarix ® (GlasxoSmithKline), grown in MA104 cells and Mock Infected MA104 cell lysate used to determine nonspecific binding were added in alternate columns to the coated microtiter plates and incubated. Following incubation on a rotating platform (60min, 37°C), the plates were washed with phosphate buffered saline + 0.05% Tween 20 (Fisher Scientific) (PBST). The reference standard, controls and samples were diluted in diluent (PBST + 1% nonfat dry milk) and were added (50 μl well) and incubated (60min, 37°C, rotating platform). The plates were washed and biotinylated goat anti-human IgA (Jackson Laboratories) was added and incubated. Plates were washed and peroxidase conjugated avidin:biotin (Vector Laboratories, Inc., Burlingame, CA) diluted in wash buffer was added and incubated. After a final wash, the substrate O-phenylenediamine (OPD) (Sigma Aldrich, St Louis, MO) was added. Development was stopped after 30 minutes with 100 μl per well of 1.0M H_2SO_4. Plates were read at 492nm on a Molecular Devices SpectraMax 190 plate reader. A standard curve was modeled using a four parameter logistic regression function in the SoftMax software for the reader. The concentration of anti-rotavirus IgA in a sample was derived by extrapolation from the reference standard curve. The reference standard is a pool of human sera collected from subjects know to be infected with rotavirus and was assigned a value of 1,000 units of anti-rotavirus IgA per mL. The lower limit of quantitation was determined during validation of the assay to be 7.5 units per mL for anti-rotavirus IgA.

Fecal sample collection

Fecal samples were collected by community health workers in infants' homes, transported in a cool box, and frozen to −20°C within 24–48 hours of collection in Navrongo, Ghana. The samples were transported in coolers with freezing cartridges or dry ice for further storage at −80°C in Accra, Ghana. All samples were stored in 3% glycerol in frost-free freezers. Routine temperature monitoring did not indicate any freeze-thaw cycles.

16S rRNA gene amplicon sequencing

16S rRNA gene amplicon sequencing was performed by extracting stool total nucleic acid from aliquots of pulverized human stool as described previously (Reyes et al., 2013). Primer selection and polymerase chain reaction were performed following previously described methods (Caporaso et al., 2011). Briefly, DNA was phenol/chloroform-extracted and amplified in triplicate with Golay-barcoded primers specific for the V4 region of the 16S rRNA gene. Amplicons were pooled and purified with 0.6 × Agencourt Ampure XP beads (Beckman-Coulter) prior to sequencing at the DNA Sequencing Innovation Laboratory at the Edison Family Center for Genome Sciences, Washington University School of Medicine using the 2 × 250-bp protocol on the Illumina MiSeq platform. All samples were processed on the same day with one batch of reagents by the same technician and sequenced on a single MiSeq run to minimize batch effects. All 16S rRNA gene sequences were uploaded to the European Nucleic Acid Archive (ENA) under project PRJEB39845.

Bacteriome analysis

An average of 35,925 sequences with standard deviation of 27,860.81 per sample were obtained from 441 samples. A total of 59 samples with fewer than 10,000 sequences were removed from subsequent analysis. Amplicon sequence variants (ASV) were selected from unrarefied data using DADA2 (Callahan et al., 2016). ASVs not assigned to the kingdom Bacteria or assigned to the family of Mitochondria, the Class of Chloroplast or the Phylum of Cyanobacteria/Chloroplast were removed from the analysis. The phyla Fusobacteria and Verrucomicrobia were removed from the analysis resulted from filtering low prevalence phyla. Following removal of those ASVs, 1,349 ASVs remained. Taxonomy was assigned using the Ribosomal Database Project (RDP) 16S rRNA gene sequence database (Cole et al., 2014). R packages including PhyloSeq were used to complete all subsequent analyses (McMurdie and Holmes, 2013). Average proportions of each phylum in the total bacteriome composition for Ghanaian infants identified as non-seroconverters (“No”) or seroconverters (“Yes”) over three doses (Dose 1, 2, 3) was used for relative abundance plot. Differences of alpha-diversity (richness and Shannon diversity) and beta-diversity (weighted UniFrac distance) between groups were evaluated using the Wilcoxon test and Permutational Multivariate Analysis of Variance (ADONIS), respectively. ASVs differentially abundant between “No” and “Yes” of serostatus groups at each dosing period were identified by comparing their regularized log transformed normalized abundances with selection criteria of adjusted p value < 0.05 and base mean (the average of the normalized counts divided by size factors taken over all samples) > 100 using DESeq2 (Love et al., 2014).

Viral-like particle preparation

Stool viral-like particles (VLP) were prepared from 308 samples as previously described (Finkbeiner et al., 2009). In brief, approximately 100-200 mg of frozen stool were resuspended in buffer and filtered through 0.45 μm filters until clarified. Clarified samples were subsequently treated with lysozyme to liberate bacterial nucleic acid followed by DNase treatment to remove non-encapsidated viral nucleic acid. Total nucleic acid (both RNA and DNA) was extracted on a COBAS AmpliPrep instrument (Roche) according to the manufacturer recommendations. Purified total nucleic acid was reverse-transcribed and PCR amplified using barcoded primers consisting of a base-balanced 16 nucleotide specific sequence and used for NEBNext library construction (New England BioLabs). Libraries were multiplexed (12 samples per flow-cell) on an Illumina MiSeq instrument (DNA Sequencing Innovation Laboratory at the Edison Family Center for Genome Sciences, Washington University School of Medicine) using the paired-end 2x250 protocol. This resulted in an average of 9.41×10^5 reads per sample (stdev = 6.76×10^5). All unprocessed virome sequences were uploaded to the European Nucleic Acid Archive (ENA) under project PRJEB39845.

Viral sequencing analysis

Unprocessed paired-end reads were processed through a multistage quality control procedure to remove primers and adapters, human and other contaminant and low-quality sequence data (Figure S2). Exact duplicate sequences were removed reserving a single copy and all remaining sequence dereplicated allowing for 4 substitutions. These high-quality/low-redundancy sequences were systematically queried against protein or genomic reference databases using MMseqs2 translated and untranslated search strategies (Figure S2) (Steinegger and Söding, 2017). Sequences assigned a eukaryotic viral taxonomic lineage were first identified using primary searches against virus sequence databases and subsequently confirmed in secondary searches using reference databases with additional non-viral taxonomic lineages. Specific viral families were selected for subsequent analysis if they were represented by more than 1,000 reads in the entire data set (0.03% of reads). Viral families were removed if < 1% of the sequences assigned to that family had low percent identity (< 70%) over short (< 50 bases) alignments. Detection of small numbers of reads from the *Virgaviridae* (n = 319 reads, plant virus), *Endornaviridae* (n = 17 reads, plant/fungal virus), *Genomoviridae* (n = 12 reads, fungal virus), *Chrysoviridae* (n = 10 reads, plant/fungal virus), *Papillomaviridae* (n = 9 reads, vertebrate virus) and *Paramyxoviridae* (n = 3 reads, vertebrate virus) families were noted; and, these were excluded from further analysis due to their overall rarity (0.009 – 0.952%). In total we obtained 2.97×10^8 paired-end reads with an average of 9.41×10^5 (sd: 6.76×10^5) per sample. Quality control procedures removed an average of 6.28×10^4 reads per sample, of which an average of 8.67×10^5 reads were clustered (allowing 4 substitutions) per sample. The resulting 3.35×10^6 reads (average of 8.67×10^5 per sample) were used for taxonomic assignment and abundance estimation. Iterative translated (nucleotide to protein) and untranslated (nucleotide to nucleotide) queries to viral protein and genomic reference databases assigned taxonomy to 1.54×10^5 and 2.70×10^4 respectively for a total of 1.80×10^5 (5.37% of quality-controlled sequence). A total of 2.52×10^6 reads (75.2%) were not classified as viral in either search (Figure S2).

Metavirome assembly

Individual sample assemblies were generated by providing kmer normalized (bbnorm, target = 20, mindepth = 2) high-quality control paired-end reads to MEGAHIT (default settings) (Li et al., 2015). A study-wide contig dictionary was generated by supplying MEGAHIT output contigs to Flye (–meta), essentially assembling the assemblies (Kolmogorov et al., 2020). Contigs were assigned taxonomy in the same way that individual reads were assigned taxonomy using iterative MMseqs2 searching as described above. Abundance of each contig per sample was determined by mapping individual sample reads to the contig dictionary generated by Flye using Kallisto (Bray et al., 2016).

Phylogenetic analysis

Multiple sequence alignment and phylogenetic tree calculation was completed using MEGA X (Kumar et al., 2018). Multiple sequence alignment of either nucleic or amino acid sequence was completed using MUSCLE (default settings) (Edgar, 2004). Evolutionary history was inferred using the maximum likelihood method and JTT matrix-based model with uniform rates (Jones et al., 1992). Initial seed tree was obtained using BioNJ (Gascuel, 1997). Tree inference heuristic used was the nearest-neighbor-interchange. Phylogeny was testing using the Bootstrap method with 100 replications.

Phageome analysis

Contigs and their abundances annotated to a phage lineage during metavirome assembly were selected for phageome analysis. Differences in alpha diversity (richness and Shannon diversity) and beta diversity (non-metric multi-dimensional scaling) between "No" and "Yes" of serostatus groups at each dosing period were evaluated using the Wilcoxon test and Permutational Multivariate Analysis of Variance (ADONIS), respectively.

QUANTIFICATION AND STATISTICAL ANALYSIS

Statistical analyses was performed as described in the figure legends and detailed in the results and corresponding method detail section. Subject per dose averages was quantified as below. Statistical significance was calculated with either Prism version 8.4.3 or R version 3.6.3. The statistical analyses did not evaluate the association between sex and bacterial or viral microbiome composition.

Subject per dose averages

For baceteriome analysis, each subject had a minimum of 1 and a maximum of 4 samples per vaccine dosing period (mean number of samples per patient: dose 1 = 1.66, dose 2 = 1.51, dose 3 = 1.43). Per taxon counts were averaged for each taxonomic lineage to integrate variance and compensate for uneven sampling. Per dose sample averaging resulted in 247 samples (dose 1: 95, dose 2: 84, dose 3: 68) which were used for all subsequent analyses. For virome analysis, each subject had a minimum of 1 and a maximum of 3 samples per vaccine dosing period (mean number of samples per patient: dose 1 = 1.49, dose 2 = 1.35, dose 3 = 1.22). Per taxon counts were averaged for each taxonomic lineage to integrate variance and compensate for uneven sampling. Per dose sample averaging resulted in 227 samples (dose 1: 84, dose 2: 78, dose 3: 69) which were used for all subsequent analyses.

Cell Host & Microbe, Volume *30*

Supplemental information

Enteric virome negatively affects seroconversion following oral rotavirus vaccination in a longitudinally sampled cohort of Ghanaian infants

Andrew HyoungJin Kim, George Armah, Francis Dennis, Leran Wang, Rachel Rodgers, Lindsay Droit, Megan T. Baldridge, Scott A. Handley, and Vanessa C. Harris

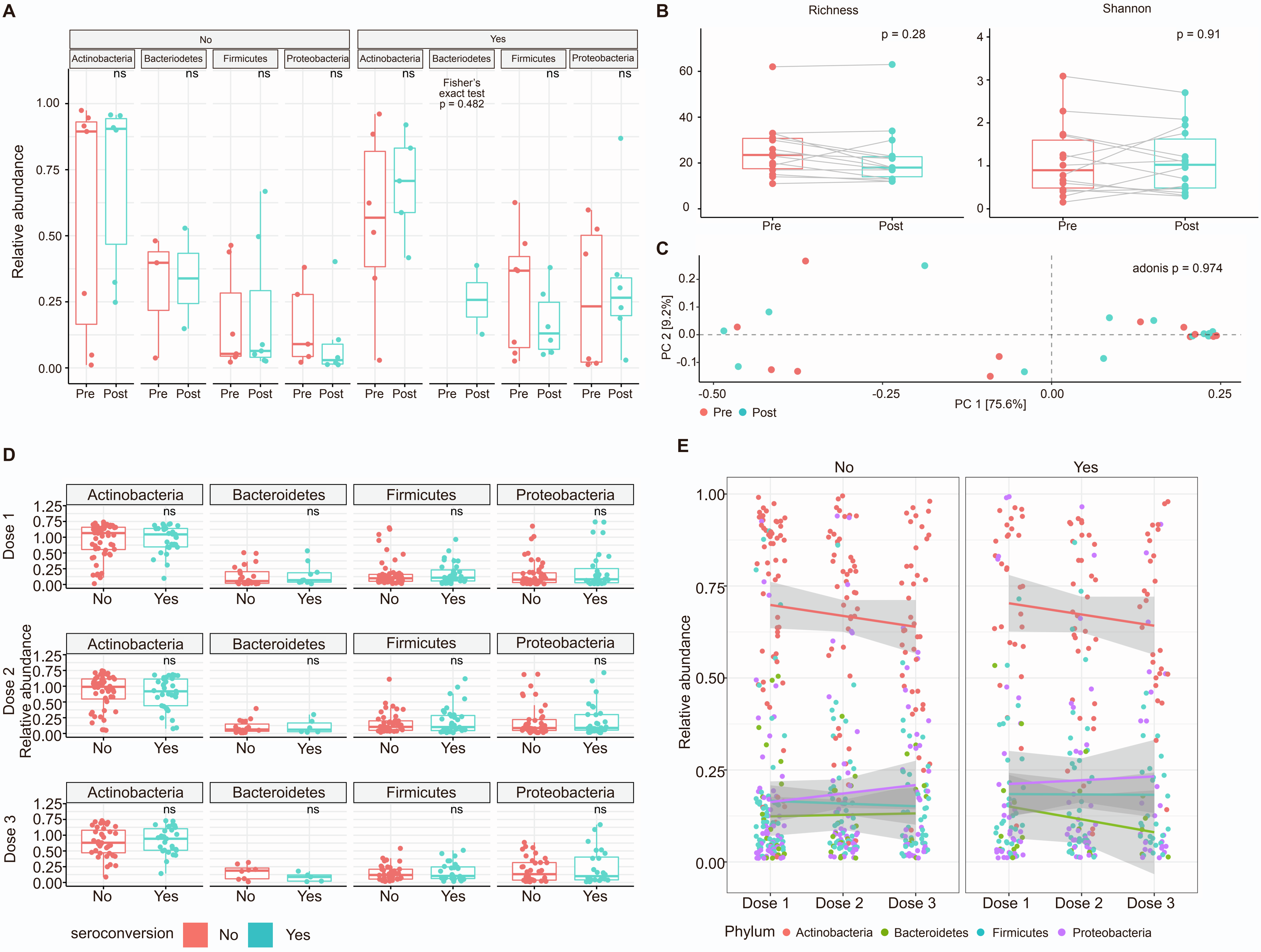

Figure S1. Phylum-level bacteriome composition analysis shows no differences between pre- and post- vaccination samples and specific to seroconversion, related to Figure 2. A) The relative abundance of bacterial phyla in 14 matched pre- and post-vaccination samples at dose 1, **B)** Bacterial richness and diversity between pre- and post-vaccination samples at dose 1, **C)** Bacterial beta diversity (weighted UniFrac distance) of pre- and post-vaccination samples at dose 1. Wilcoxon test and Permutational Multivariate Analysis of Variance (ADONIS) were used to compare between serostatus groups in alpha- and beta-diversity analyses, respectively. **D)** The relative abundance of bacterial phyla in Ghanaian infants identified as non-seroconverters ("No") and seroconverters ("Yes") at three doses (Dose 1, 2, 3) and **E)** change over time. Lines depict the linear model while greyed areas indicate the 95% confidence level interval of the model. Statistical comparison between two serostatus groups was performed using Wilcoxon test (ns = not significant). n = 148 averaged non-seroconverter samples (Dose 1: 60, Dose 2: 49, Dose 3: 39) and 99 averaged seroconverter samples (Dose 1: 35, Dose 2: 35, Dose 3: 29).

Dose	ASV	lfc	lfcse	adj p-value	Sequence	Kingdom	Phylum	Class	Order	Family	Genus	Species	Pearson's rho
Dose 1	3	2.845	0.736	3.63E-03	GCAAGCGTTAATCGGAATT	Bacteria	Proteobacteria	Gammaproteobacteria	Enterobacteriales	*Enterobacteriaceae*	NA	NA	DA
Dose 2	4	2.640	0.632	5.99E-04	CCGAGCGTTGTCCGGATTT	Bacteria	Firmicutes	Bacilli	Lactobacillales	*Streptococcaceae*	*Streptococcus*	NA	DA
Dose 2	5	9.194	2.326	1.18E-03	CCGAGCGTTGTCCGGATTT	Bacteria	Firmicutes	Bacilli	Lactobacillales	*Streptococcaceae*	*Streptococcus*	NA	DA
Dose 2	6	-9.932	2.118	8.33E-05	CCGAGCGTTGTCCGGATTT	Bacteria	Firmicutes	Bacilli	Lactobacillales	*Streptococcaceae*	*Streptococcus*	NA	DA
Dose 2	45	6.292	1.234	2.08E-05	GCAAGCGTTATCCGGATTT	Bacteria	Firmicutes	Bacilli	Lactobacillales	*Lactobacillaceae*	*Lactobacillus*	*fermentum*	DA
Dose 3	3	3.047	0.865	1.39E-02	GCAAGCGTTAATCGGAATT	Bacteria	Proteobacteria	Gammaproteobacteria	Enterobacteriales	*Enterobacteriaceae*	NA	NA	DA
Dose 3	32	2.235	0.716	3.91E-02	GCGAGCGTTGTCCGGAATT	Bacteria	Actinobacteria	Actinobacteria	Actinomycetales	*Micrococcaceae*	*Rothia*	*mucilaginosa*	DA
Dose 3	92	0.995	0.512	4.84E-03	GCGAGCGTTATCCGGATTC	Bacteria	Actinobacteria	Actinobacteria	Coriobacteriales	*Coriobacteriaceae*	*Eggerthella*	*lenta*	0.453

lfc log2(Fold change)
lfcse log2(Fold change Stardard Error)
NA Taxonomy not assigned
DA Discrete analysis

Table S1. Summary table of bacterial markers identified by DESeq2 analysis at each dosing period, related to Figure 2. Identification of bacterial markers was performed using DESeq2 and multiple Pearson's correlation analyses with cut-off threshold of adjusted p value < 0.05. Adjusted p values were attained for each bacterial marker by performing Wald test followed by correction using Benjamini and Hochberg method for DESeq2 and Pearson's correlation analysis followed by correction using Bonferroni method. Log2(Fold change) and log2(Fold change Standard Error) show the seroconverter ("Yes") over non-seroconverter ("No") fold change and its standard error for the abundance of each bacterial marker. Black texts indicate markers selected from DESeq2 analyses and gray texts indicates a marker selected from multiple Pearson's correlation analysis.

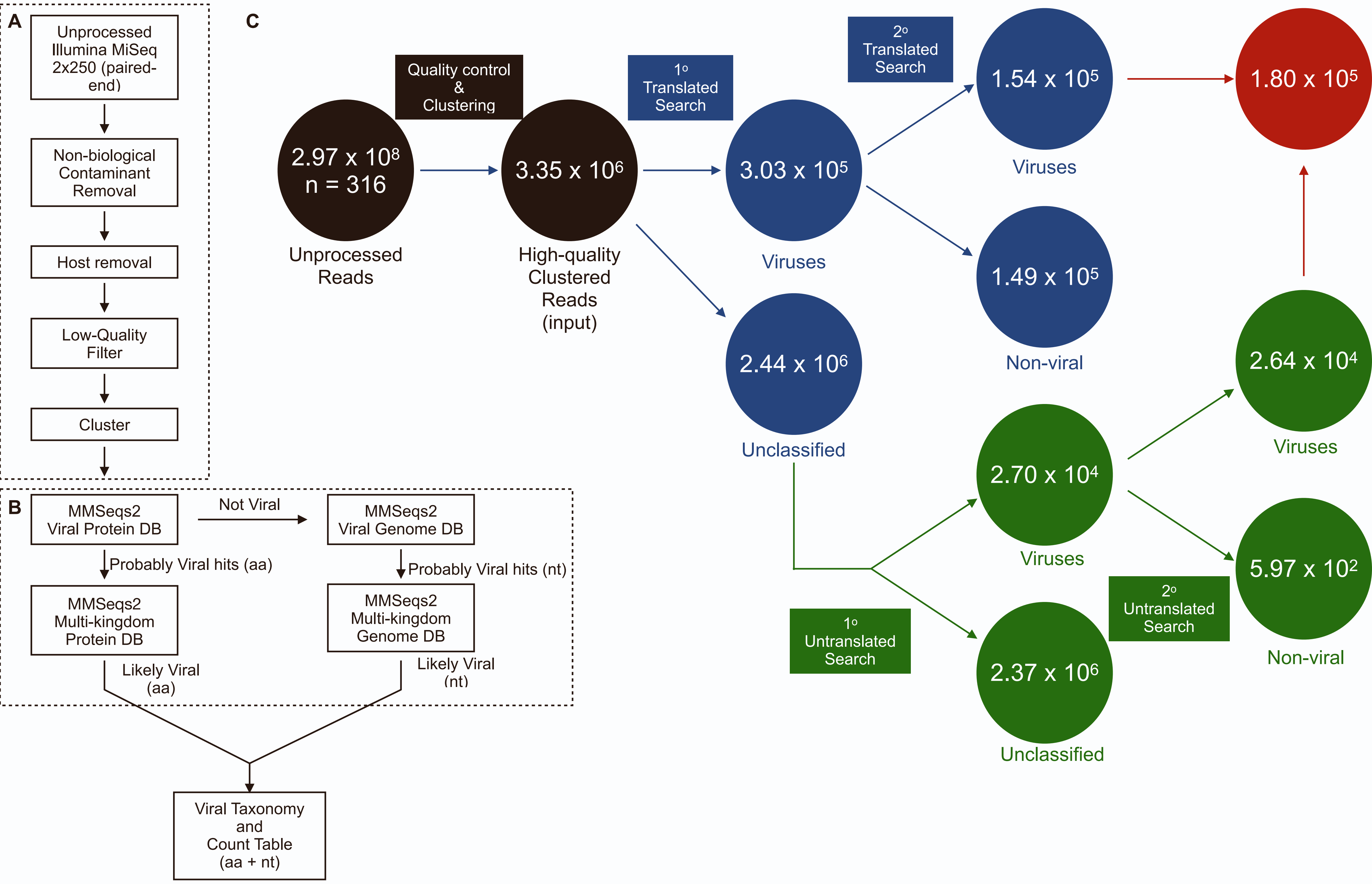

Figure S2. Viral taxonomic analysis of Illumina reads, related to STAR Methods. High-quality reads from 316 samples (black circle) were quality controlled to remove non-biological sequence (primer, adapter), host and low-quality sequence (Figure S2). These high-quality sequences were 6-frame translated and queried (blue path) against a viral protein sequence database (UniProt viral proteins clustered at 99% identity) followed by a secondary translated query of all potential viral sequences against a universal protein sequence database (UniClust30) to remove mis-classified viral sequences (Mirdita et al., 2017). Sequences that were not classified in the translated search were queried (green path) against a viral reference genome database (all RefSeq viral genomes plus their nearest neighbors). Potential viral sequences were subsequently queried against a bacterial plus viral genome database to remove sequences ambiguous to bacteria and virus. The translated and untranslated query sequences assigned to a viral taxonomic lineage were combined into a final (red circle) sequence table.

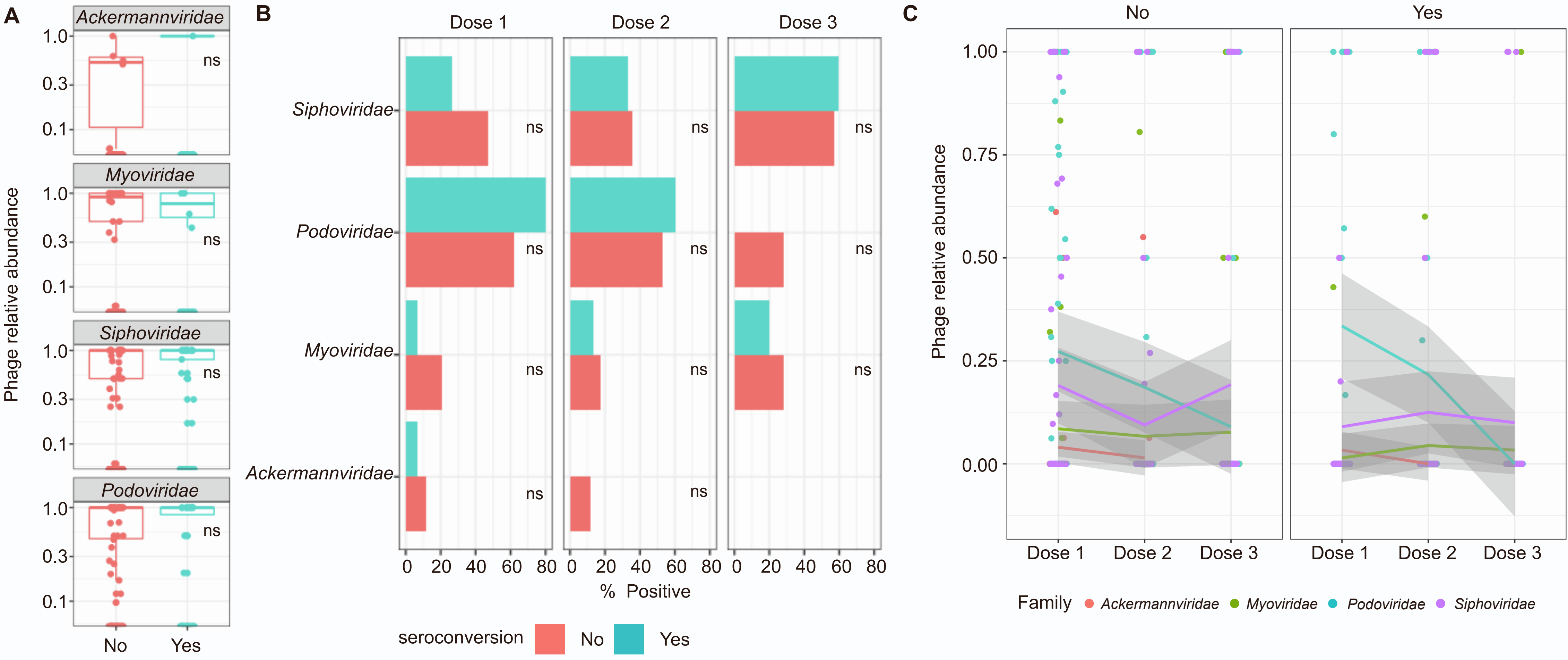

Figure S3. Phage composition does not associate with seroconversion when analysed at the family-level, related to Figure 3. A) phage relative abundance in Ghanaian infants identified as non-seroconverters ("No") and seroconverters ("Yes") at three doses (Dose 1, 2, 3). **B)** The percent positive of phage families at three doses (Dose 1, 2, 3). **C)** phage relative abundance change over time. Statistical comparison between two serostatus groups was performed using Wilcoxon test (ns = not significant). Lines depict the linear model while greyed areas indicate the 95% confidence level interval of the model. n = 216 non-seroconverter samples (Dose 1: 54, Dose 2: 84, Dose 3: 78) and 162 seroconverter samples (Dose 1: 30, Dose 2: 72, Dose 3: 60).

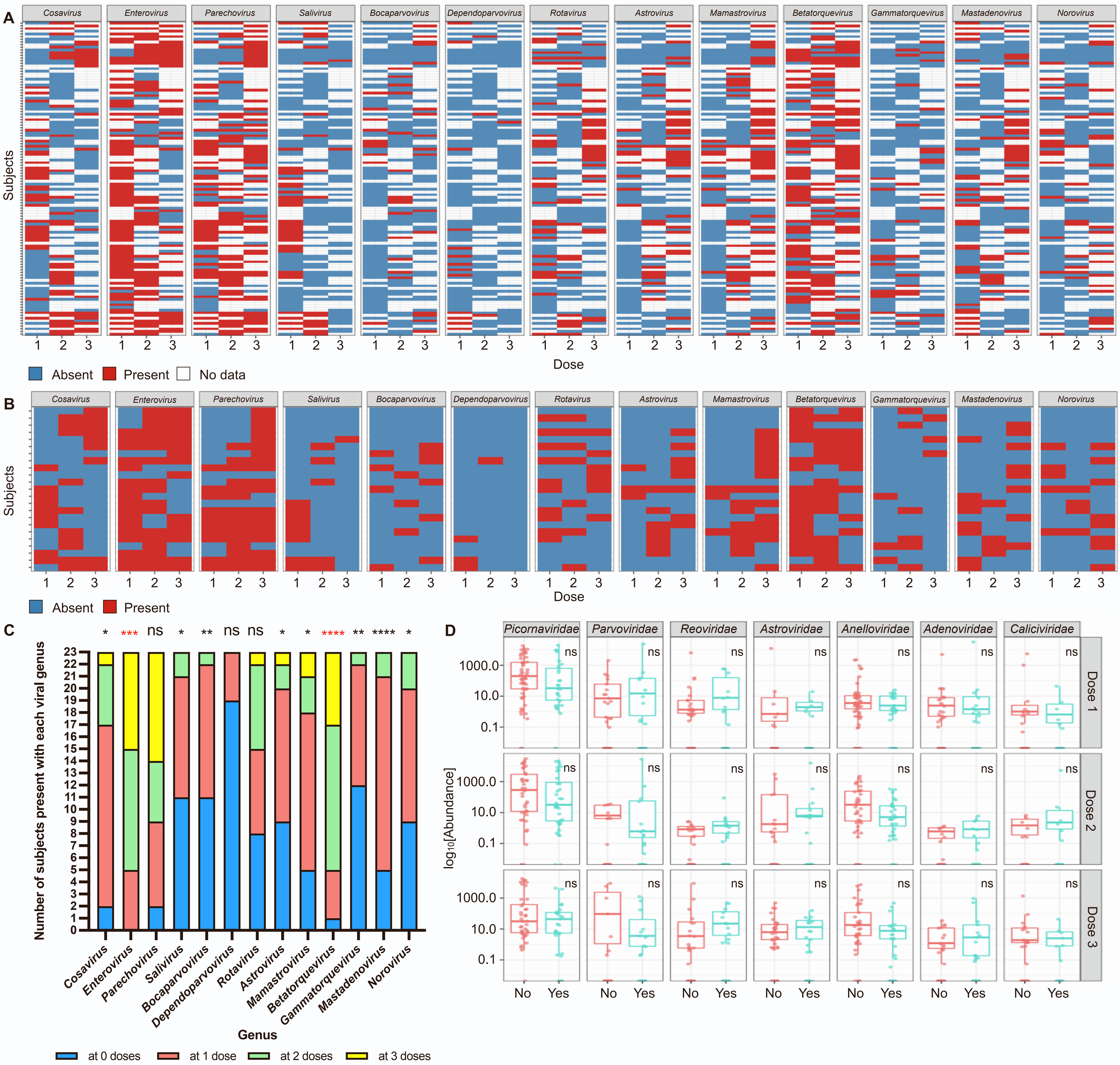

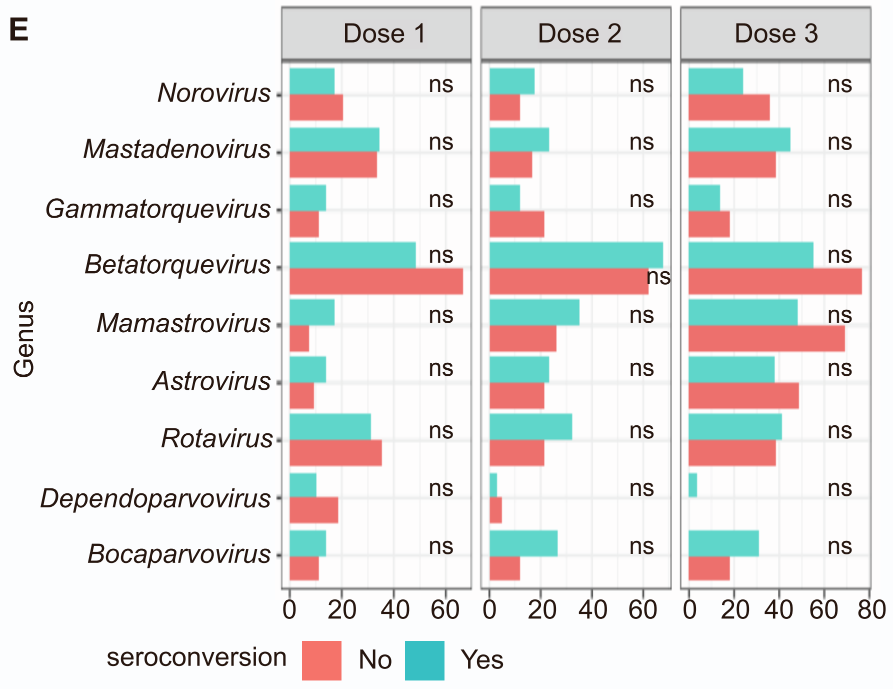

Figure S4. Viral persistence varies per viral family and seroconversion does not associate with mean abundance at the family level or percent positivity at the viral genera level, related to Figure 5. Presence and absence of each viral genus over time in **A)** all infants and **B)** a subset of 23 infants with data available at every dose. **C)** Number of infants with presence of each viral genus at one or more doses in the subset of 23 infants. Viral persistence per genus was defined as a significantly higher number of infants with presence of a viral genus at more than 1 dose as compared to at 1 dose alone and tested using Fisher's exact test (*p < 0.05, **p < 0.01, ***p < 0.001, ****p < 0.0001, ns = not significant). Red asterisk represents a significantly higher number of infants with virus present at greater than 1 dose compared to 1 dose alone and black asterisk represents a significantly higher number of infants for 1 dose alone compared to more than 1 dose. **D)** The log transformed mean abundance of eukaryotic viral families in Ghanaian infants identified as non-seroconverters ("No") and seroconverters ("Yes") at three doses (Dose 1, 2, 3). Statistical comparison between two serostatus groups was performed using Wilcoxon test (ns = not significant). n = 135 averaged non-seroconverter samples (Dose 1: 54, Dose 2: 42, Dose 3: 39) and 92 averaged seroconverter samples (Dose 1: 29, Dose 2: 34, Dose 3: 29). E) The presence (% of samples detected) of each viral genera with respect to serostatus.

Figure S5

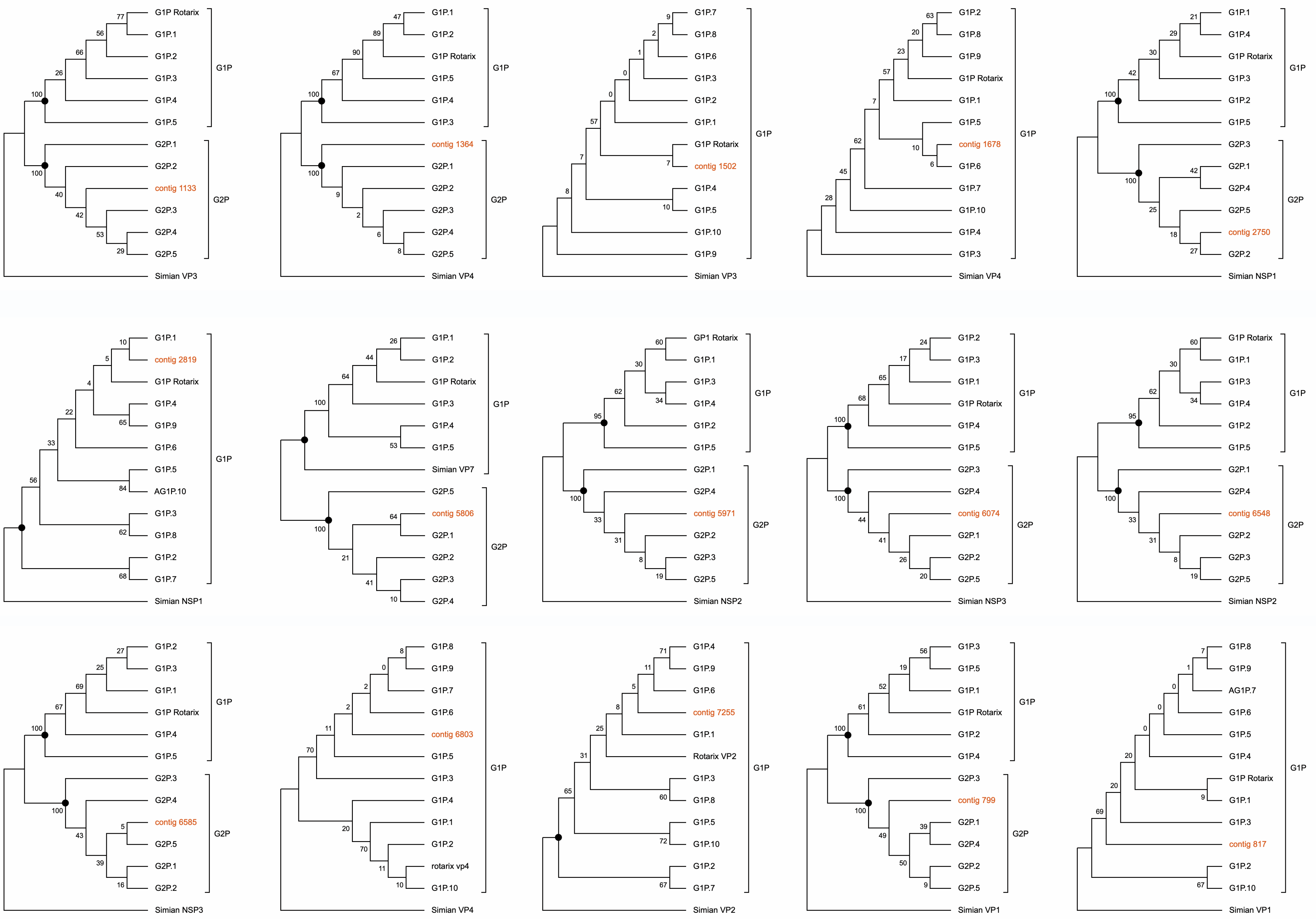

Figure S5. Strain Classification of rotavirus contigs, related to Figure 5. Phylogenetic analysis of rotavirus contigs (n = 15). Contigs were aligned to their top-10 hits resulting from a blastp search against NCBI nr. If both G1P and G2P were present in the top-10, then the top-5 G1P and G2P hits were collected for multiple sequence alignment. If only G1P or G2P were present, then the top 10 from that group was collected. Multiple sequence alignments to the top-10 were trimmed and the resulting alignment used for phylogenetic tree construction using maximum-likelihood inference (GTR+gamma). The Simian version of the aligned protein was used as an outgroup. Branch stability was determined using 100 bootstrap replicates. Metagenome assembled contig is indicated in orange. G1P and G2P brackets were applied when a branch was separated by a bootstrap > 95.

Figure S6

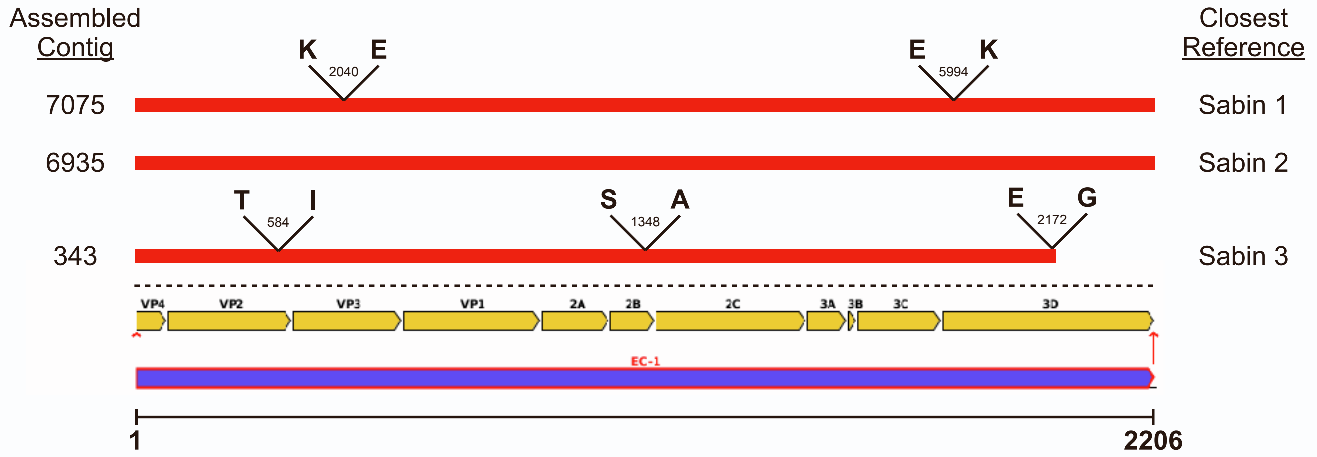

Figure S6. Assembled contig of oral polio vaccine strains, Sabin 1, Sabin 2 and Sabin 3, related to Figure 6. Assembly recovered a Sabin 1 contig containing a full-length polyprotein with two amino acid mutations (c.2040K>E in VP3 and c.5994E>K in RD3) when compared to published Sabin 1 genomes. The recovered Sabin 2 contig was full-length and identical to reference Sabin 2 polyprotein. The recovered Sabin 3 contig was 3’ truncated by 32 amino acids of the 3D (RNA directed RNA polymerase) and had 3 mutations (c.584T>I in VP2, c.1348S>A in protein B and c.2172E>G in 3D).
